# 4-Hydroxy-3-methyl-2(1*H*)-quinolone, originally discovered from a *Brassicaceae* plant, produced by a soil bacterium of the genus *Burkholderia* sp.: determination of a preferred tautomer and antioxidant activity

**DOI:** 10.3762/bjoc.16.124

**Published:** 2020-06-26

**Authors:** Dandan Li, Naoya Oku, Yukiko Shinozaki, Yoichi Kurokawa, Yasuhiro Igarashi

**Affiliations:** 1 Biotechnology Research Center and Department of Biotechnology, Toyama Prefectural University, 5180 Kurokawa, Imizu, Toyama 939-0398, Japan; 2 National Institute of Technology, Toyama College, 13 Hongo-machi, Toyama City, Toyama 939-8630, Japan; 3 Department of Bioscience and Biotechnology, Fukui Prefectural University, Eiheiji-cho, Fukui, Japan

**Keywords:** antioxidant, *Burkholderia* sp, quinolone, soil bacterium, Zn^2+^ enrichment culture

## Abstract

4-Hydroxy-3-methyl-2(1*H*)-quinolone (**1**), a molecule known for a long time and recently discovered from a *Brassicaceae* plant *Isatis tinctoria* without providing sufficient evidence to support the structure, was isolated from a fermentation extract of *Burkholderia* sp. 3Y-MMP isolated from a soil by a Zn^2+^ enrichment culture. Detailed spectroscopic analyses by MS and NMR, combined with ^13^C chemical shift comparison with literature values of the related compounds and a synthetic preparation of **1**, allowed its first full NMR characterization and identification of 2-quinolone but not 2-quinolinol (**2**) as the preferred tautomer for this heterocyclic system. While the metal-chelating activity was negligible, compound **1** at 10 μM, a concentration lower than that in liquid production cultures, quenched hydroxy radical-induced chemiluminescence emitted by luminol by 86%. Because some *Burkholderia* species are pathogenic to plants and animals, the above result suggests that **1** is a potential antioxidant to counteract reactive oxygen species-based immune response in the host organisms.

## Findings

4-Hydroxy-2(1*H*)-quinolone (**3**) is a unique structural motif mostly found in alkaloids from rutaceous plants (family *Rutaceae*) [1–2]. This motif has several tautomeric forms including 2,4-dihydroxyquinoline (**4**) [3–5], although which form to be taken seems not always be correctly identified in some of the studies [6–8]. Currently, 229 compounds are known to contain this unit as a part or a whole of the structure, among which only 12 originated from organisms other than rutaceous plants [9]. Examples from microbes include chymase inhibitors SF2809-I to VI from an actinomycete of the genus *Dactylosporangium* [10], a quorum sensing signaling molecule 2,4-dihydroxyquinoline (DHQ, **4**) from Gram-negative bacteria *Pseudomonas aeruginosa* and *Burkholderia thailandensis*, [7], and 4-*O*-β-ᴅ-glucopyranosyl-2,3,4-trihydroxyquinoline (**5**) from an ascomycete of the genus *Alternaria* [8].

The genus *Burkholderia* sensu lato, within the class *Betaproteobacteria*, represents a polyphyletic group of bacteria, which undergoes reclassification into several lineages [11]. Members of this group are basically free-living aerobes inhabiting soil and freshwater, but some are also found in the tissues of animals, plants, or fungi as pathogens or beneficial symbionts [12]. Not only as the subjects of human/animal health care and plant pathology [13], but they are now gathering significant attention as an emerging source of bioactive molecules. Many new structure classes, even after being spun off as a new genus from *Pseudomonas* in 1992 [14], have been discovered from this group, which, along with their large genomes comparable to those of actinomycetes or myxobacteria, demonstrate a higher capacity of secondary metabolism [15].

**Figure 1:**
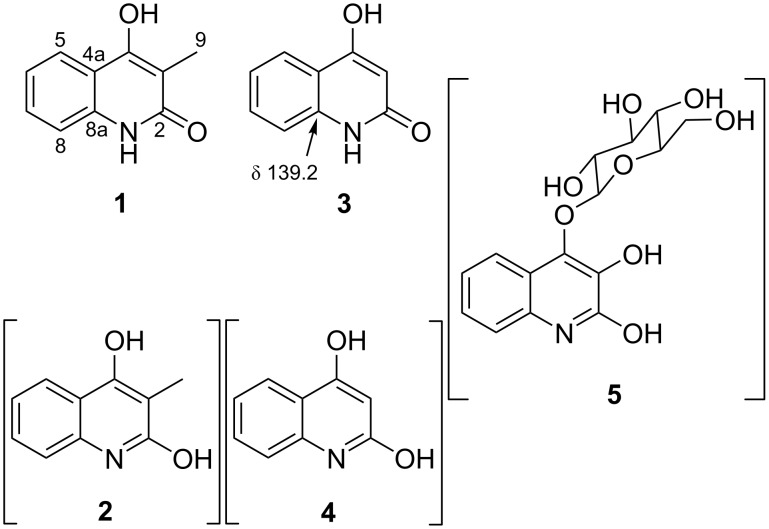
Structures of compounds **1**–**5**.

In the course of our continuing studies on bioactive metabolites of less studied bacterial taxa [16], *Burkholderia* sp. 3Y-MMP, isolated from soil by an exhaustive enrichment culture under Zn^2+^-load, was selected for a detailed chemical study, which resulted in the isolation of 4-hydroxy-3-methyl-2(1*H*)-quinolone (**1**, Figure 1). This compound was recently reported from the root of woad (*Isatis tinctoria*, family *Brassicaceae*) with no details of structure characterization [17]. Herein we describe the isolation, unequivocal structure characterization, and antioxidant activity of compound **1**.

The producing strain 3Y-MMP was cultured in King’s B medium [18] for 4 days and the production culture was extracted with 1-BuOH. The butanolic extract was partitioned between CH_2_Cl_2_ and 60% MeOH, and the latter layer was flash-chromatographed on ODS followed by reversed-phase HPLC to yield **1** (5.2 mg) with sufficient purity for structure characterization.

The molecular formula of **1** was determined to be C_10_H_9_NO_2_ based on a sodium adduct molecular ion peak at *m/z* 198.0525 observed by a HRESITOFMS measurement (calcd 198.0526). The broad IR absorption band around 3100 cm^−1^ and an intense peak at 1600 cm^−1^ indicated the existence of hydroxy and aromatic groups, respectively.

**Figure 2:**
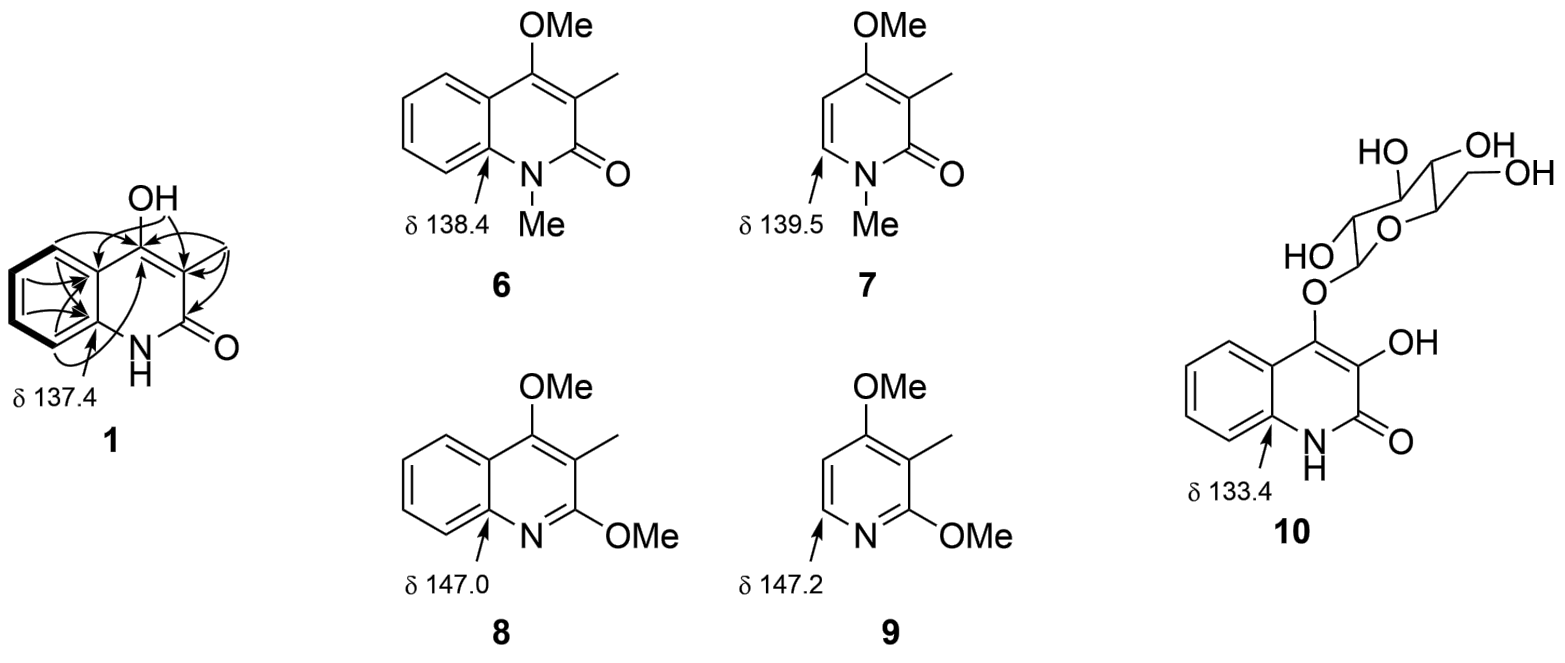
COSY-deduced spin-system (bold lines) and key HMBC correlations (arrows) for compound **1**, and structures for compounds **6**–**10** with a ^13^C chemical shift at the C8a position.

The ^1^H and ^13^C NMR spectra in DMSO-*d*_6_ displayed 6 and 10 resonances, respectively, and by combining with the results of ^1^H,^1^H coupling constants and COSY and HSQC spectroscopic analysis, following 8 molecular pieces were revealed: a consecutive four aromatic methines (δ_C_ 129.8, 122.7, 121.2, 115.0; δ_H_ 7.85, 7.41, 7.23, 7.12), two heteroatom-substituted nonprotonated sp^2^ carbons (δ_C_ 164.0 and 157.4), three sp^2^ nonprotonated carbons (δ_C_ 137.4, 115.8, and 106.9), an allylic methyl group (δ_C_ 9.6/δ_H_ 1.98 s), and a singlet exchangeable proton (δ_H_ 11.30). The four methine unit (C5–C6–C7–C8) was connected to the two quaternary carbons (δ_C_ 137.4 and 115.8: C8a and C4a) to form a disubstituted benzene ring by HMBC correlations H5/C8a, H6/C4a, H7/C8a, H8/C4a, and H8/C8a. On the other hand, the remaining parts were assembled into a C_4_ enol-amidyl or enol-imidic acyl unit based on HMBC correlations from the methyl proton H_3_9 to the three nonprotonated carbons C4 (δ 157.4), C3 (δ 106.9), and C2 (δ 164.0). Connection of this unit to C4a of the benzene ring was implied by an HMBC correlation from H_3_9 to C4a, and correlations from the exchangeable proton to C4a and C3 supported this linkage as well as hydroxylation at the benzylic position. Finally, the chemical shift of C8a at 137.4 ppm was in favor of *N*-substitution, and comparison with the literature values from 4-methoxy-1,3-dimethyl-2(1*H*)-quinolone (**6**, δ 138.4) [19], *N*-methyl-2-pyridone **7** (δ 139.5) [20], 2,4-dimethoxy-3-methylquinoline (**8**, δ 147.0) [21], and 2-methoxypyridine **9** (δ 147.2) [22] supported a 2-quinolone form **1** but not 2-quinolinol **2** (Figure 2, Table 1). The same structure was synthesized from diethyl malonate and aniline (see Supporting Information File 1 for full experimental data), which substantiated this assignment.

**Table 1:** ^1^H (500 MHz) and ^13^C (125 MHz) NMR data of compound **1** in DMSO-*d*_6_ (297 K).

	**1**		

No.	^13^C	^1^H multiplicity, (*J* in Hz), integration	HMBC (→^13^C)

1^a^			
2	164.0		
3	106.9		
4	157.4		
4-O*H*		11.30 brs, 1H	3, 4a
4a	115.8		
5	122.7	7.85 dd (7.9, 1.0), 1H	4, 7, 8a
6	121.2	7.12 ddd (7.9, 7.2, 0.7), 1H	4a, 5, 7, 8, 8a
7	129.8	7.41 ddd (8.1, 7.2. 1.2), 1H	5, 8, 8a
8	115.0	7.23 d (8.1), 1H	4, 4a, 6, 7, 8a
8a	137.4		
9	9.6	1.98 s, 3H	2, 3, 4, 4a

^a^Signal for amide proton not observed.

Although compound **1** has repeatedly been synthesized since 1921 [23] and enumerated chemical shifts for ^1^H and ^13^C resonances were available [24], one-on-one assignments of the resonances to each structural part have not been made until this work. In addition, HMBC correlations from the enol proton and the comparison of the chemical shift of the carbon adjacent to the nitrogen with the literature values unequivocally determined 2-quinolone to be a preferred tautomer of this heterocyclic system. The same C8a carbons of compounds **4** and **5** resonate at 139.2 [25] and 133.4 ppm [8], respectively (Figure 1 and Figure 2), which indicates that both also exist as 2-quinolone and hence should more precisely be called as 4-hydroxy-2(1*H*)-quinolone (4HQ, **3**) and 4-*O*-β-ᴅ-glucopyranosyl-3,4-dihydroxy-2-quinolone (**10**), respectively.

Though not alkylated, the close structural similarity to **3** suggests that **1** is also a member of the 2-alkyl-4-quinolone class signaling molecules/antibiotics known from *Pseudomonas aeruginosa* and some *Burkholderia* species [26–27]. Quinolones of this class are classified into two lineages, those with or without a 3-methyl group, and the former lineages are unique to *Burkholderia* producers [28]. These metabolites are shown to be biosynthesized by head-to-head condensation of anthranilate and β-ketoacylate precursors, followed by a modification at C3 or nitrogen by putative monooxygenases or methyltransferase [27]. Entry of malonate as the acylate precursor into this pathway is proposed to yield **3** (**4** in the original literature) [29]. Thus, **1** is very likely to be biosynthesized by the same mechanism followed by additional methylation on C3.

Compound **1** is reportedly inhibitory to *Mycobacterium tuberculosis* H37Ra at IC_90_ 6.8 μM while weakly cytotoxic to MRC-5 human lung-derived fibroblasts with GI_50_ 84.7 μM [30]. It did not inhibit the production of nitric oxide in RAW 264.7 murine macrophage-like cells [31]. In our hands, **1** was inactive against any of the tested strains including *Staphylococcus aureus* FDA209P JC-1 (Gram-positive bacterium), *Rhizobium radiobacter* NBRC14554, *Ralstonia solanacearum* SUPP1541, *Tenacibaculum maritimum* NBRC16015 (Gram-negative bacteria), *Candida albicans* NBRC0197, and *Saccharomyces cerevisiae* S100 (yeasts).

Oxidative burst, which is a transient production of massive reactive oxygen species (ROS), is implemented in eukaryotic cells, including protists [32], as an innate immune response to deactivate foreign substances or as part of phagocytic digestion of internalized nutrients [33]. Pathogenic microbes are equipped with a multitude of strategies to circumvent host immunity [33], among which redox enzymes and antioxidants are the direct countermeasures to neutralize the toxicity of ROS [34]. Limited examples of antioxidants include catecholamine melanin from a fungus *Cryptococcus neoformans* [35], 1,8-dihydroxynaphthalene melanin from fungi *Wangiella dermatitidis* and *Alternaria alternata* [36], staphyloxanthin from a firmicute *Staphylococcus aureus* [37], vitamin B_6_ from fungi *Cercospora nicotianae*, [38] and *Rhizoctonia solani* [39], and a melanin-like pigment from *Burkholderia cenocepacia* [40].

**Figure 3:**
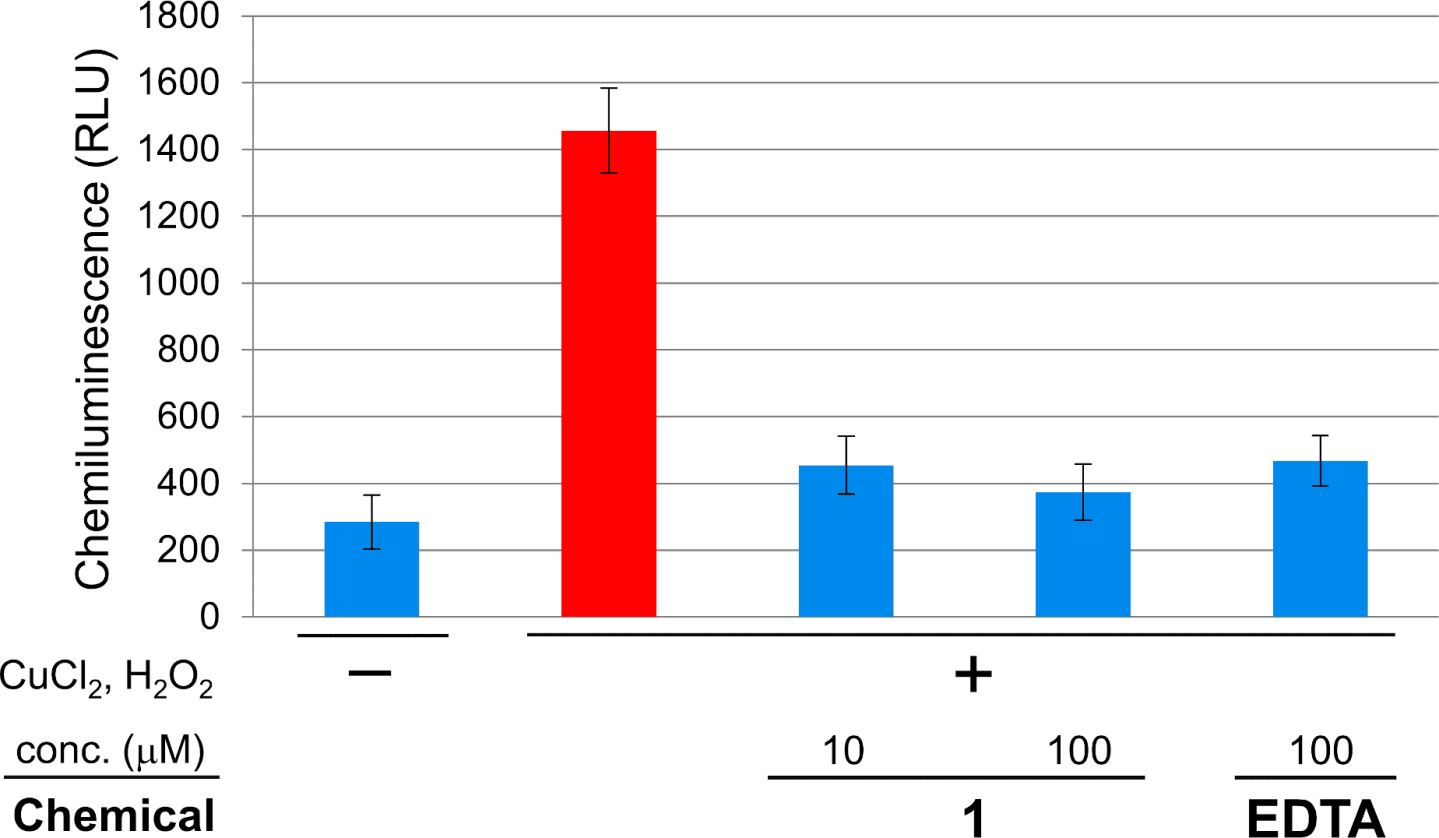
Extinction of luminol chemiluminescence by **1**.

The antioxidant activity of **1** was evaluated using the luminol chemiluminescence extinction assay [41–42]. This assay quantifies the presence of the most detrimental ROS, hydroxy radical [43–44], as intensity of luminescence emitted by oxidation of luminol. Compound **1** at 10 μM, which is lower than a concentration in production liquid cultures (15 μM), decreased luminescence to 14% of the control reaction (Figure 3). Because the Fenton reaction catalyzed by Cu^2+^ was used to generate the hydroxy radicals, entrapment of Cu^2+^ by **1** was initially suspected as the mechanism of chemiluminescence inhibition. However, this speculation was ruled out by a titration experiment using Chrome Azurol S-Fe^3+^ (CAS) [45], which required a 1600 times higher concentration for metal-chelation. Thus, compound **1** was found to be another example of the antioxidant from *Burkholderia*. Detailed studies on the antioxidation mechanism of **1** is now underway.

## Experimental

### General experimental procedures

UV and IR spectra were obtained on a Hitachi U-3210 and a Perkin Elmer Spectrum 100, respectively. NMR spectra were collected on a Bruker AVANCE 500 spectrometer in DMSO-*d*_6_ and CDCl_3_ referenced at δ_H_/δ_C_ 2.49/39.8 and 7.27/77.0, respectively. HRESITOFMS were recorded on a Bruker micrOTOF focus mass spectrometer. Chemiluminescence was measured on a Molecular Devices SpectraMax M2 microplate reader.

### Microorganism

*Burkholderia* sp. 3Y-MMP was isolated from a soil sample collected in Toyama, central Japan, in June 2015 by a procedure similar to that described in [46]. One mM of ZnCl_2_, instead of CoCl_2_, was used as a selection pressure during the initial exhaustive enrichment culture stage. The 16S rDNA sequence of strain 3Y-MMP was determined by a DNA analysis service (Tsuruga Bio, Toyobo Co. Ltd., Osaka, Japan) using a primer set 10F (5′-GTTTGATCCTGGCTCA-3′) and 800R (5′-TACCAGGGTATCTAATCC-3′). A partial sequence with a length of 800 bp (accession number LC508727) thus read was queried against the Basic Local Alignment Search Tool program (BLAST) available at the DNA Data Bank of Japan (DDBJ) website, which reported 99.9% homology to *Burkholderia cepacia* strain N1_1_43 (accession number MN691134). This strain will be deposited in NBRC once it resumes services, which is currently suspended due to a nation-wide State of Emergency regarding COVID-19 declared on April 16 by the Government of Japan.

### Fermentation and isolation

A cell mass of *Burkholderia* sp. 3Y-MMP, scraped off from an agar plate, was inoculated into 500 mL K-flasks each containing 100 mL King’s B medium composed of peptone 2%, glycerin 1%, K_2_HPO_4_ 0.41%, and MgSO_4_·7H_2_O 0.15%. The production cultures thus made were rotary shaken at 200 rpm at 30 °C for 4 days. After fermentation, an equal amount of 1-butanol was added to each flask, shaken for additional 1 h, and then centrifuged at 6000 rpm. The butanol layer was collected and dried in vacuo to give a solid (2.7 g) from a 2 L culture. The extract was partitioned between 60% aqueous MeOH and CH_2_Cl_2_, and the former layer was fractionated on ODS eluted sequentially with a step gradient of MeCN/0.1% HCOOH mixed in ratios of 2:8, 3:7, 4:6, 5:5, 6:4, 7:3, and 8:2, respectively. A fraction eluted by 30% MeCN was evaporated to provide 69.4 mg of a solid, which was purified by HPLC on an ODS column (Cosmosil AR-II, 1 × 25 cm) eluted with 16% MeCN containing 0.1% HCO_2_H at a flow rate of 4 mL/min, which yielded **1** (5.2 mg, *t*_R_ 31.3 min) with sufficient purity for NMR-based structure characterization.

4-Hydroxy-3-methyl-2(1*H*)-quinolone (**1**): UV (MeOH) λ_max_ nm (ε): 312 (2300), 226 (12000); IR (ATR) ν_max_: 3268, 3186, 2958, 2927, 1595, 1486, 1387, 1354, 1243, 1026, 772, 761, 692, 664 cm^−1^; HRESITOFMS (*m*/*z*): [M + Na]^+^ calcd for C_10_H_9_NNaO_2_, 198.0526, found: 198.0525 ; ^1^H and ^13^C NMR data are shown in Table 1.

### Evaluation of Fe^3+^ binding activity

The iron-binding activity was evaluated by the CAS assay developed by Schwyn and Neilands [45]. Compound **1** (2.5 mg) in DMSO (20 μL) was mixed with a blue-colored CAS stock solution (50 μL) and further brought up to 100 μL with H_2_O (final concentration of **1**: 160 mM). After 10 min at an ambient temperature, the solution turned orange due to the loss of Fe^3+^ from the indicator CAS dye, indicating positive to the iron-binding ability of **1**. A prolonged reaction caused biphasic separation of the mixture.

### Antimicrobial assay

The antimicrobial activity was evaluated by the method described previously [16].

### Antioxidant assay

The antioxidant activity was evaluated by the method described in [41]. Briefly, luminol (10 μM), H_2_O_2_ (1000 μM), and vehicle solvent with or without test compounds were mixed in 50 mM boric acid/sodium hydroxide buffer (pH 9.0). To this mixture was added CuCl_2_ (100 μM) to initiate the Fenton reaction, and after 5 min of incubation, the chemiluminescence at 500 nm was recorded on a microplate reader. The experiments were run in triplicate, and the mean ratio of light extinction was expressed as the potency of antioxidant activity.

## Supporting Information

File 1 Synthetic procedure of **1**, UV, IR, ^1^H NMR, ^13^C NMR, COSY, HSQC, HMBC spectra for natural and synthetic **1**, and UV, IR, ^1^H NMR, ^13^C NMR spectra for synthetic intermediates.
