# Supplementary material for: 4-Hydroxy-3-methyl-2(1H)-quinolone, originally discovered from a Brassicaceae plant, produced by a soil bacterium of the genus Burkholderia sp.: determination of a preferred tautomer and antioxidant activity
Source: Beilstein J Org Chem. 2020 Jun 26;16:1489–94. doi: 10.3762/bjoc.16.124 (PMC7323622; doi:10.3762/bjoc.16.124)
Supplement: File 1 — Synthetic procedure of 1, UV, IR, 1H NMR, 13C NMR, COSY, HSQC, HMBC spectra for natural and synthetic 1, and UV, IR, 1H NMR, 13C NMR spectra for synthetic intermediates. [file Beilstein_J_Org_Chem-16-1489-s001.pdf]

BEILSTEIN JOURNAL OF ORGANIC CHEMISTRY

Supporting Information

for

4-Hydroxy-3-methyl-2(1*H*)-quinolone, originally discovered from a *Brassicaceae* plant, produced by a soil bacterium of the genus *Burkholderia* sp.: determination of a preferred tautomer and antioxidant activity

Dandan Li, Naoya Oku, Yukiko Shinozaki, Yoichi Kurokawa and Yasuhiro Igarashi

Beilstein J. Org. Chem. **2020,** *16,* 1489–1494. doi:10.3762/bjoc.16.124

Synthetic procedure of 1, UV, IR, ^{1}H NMR, ^{13}C NMR, COSY, HSQC, HMBC spectra for natural and synthetic 1, and UV, IR, ^{1}H NMR, ^{13}C NMR spectra for synthetic intermediates

License and Terms: This is a supporting information file under the terms of the Creative Commons Attribution License (http://creativecommons.org/licenses/by/4.0). Please note that the reuse, redistribution and reproduction in particular requires that the authors and source are credited.
The license is subject to the *Beilstein Journal of Organic Chemistry* terms and conditions: (https://www.beilstein-journals.org/bjoc)

Table of contents

Synthetic procedure of **1** S1

4-Hydroxy-3-methyl-2(1*H*)-quinolone (**1**)

UV spectrum of natural **1** S3
IR spectrum of natural **1** S4
UV spectrum of synthetic **1** S5
IR spectrum of synthetic **1** S6
^{1}H NMR spectra of natural (upper) and synthetic (lower) **1** (DMSO-d_6, 297 K, 500 MHz) S7
^{13}C NMR spectra of natural (upper) and synthetic (lower) **1** (DMSO-d_6, 297 K, 125 MHz) S8
COSY spectrum of natural **1** (DMSO-d_6, 297 K, 500 MHz) S9
HSQC spectrum of natural **1** (DMSO-d_6, 297 K, 500 MHz) S10
HMBC spectrum of natural **1** (DMSO-d_6, 298 K, 500 MHz) S11
COSY spectrum of synthetic **1** (DMSO-d_6, 298 K, 500 MHz) S12
HSQC spectrum of synthetic **1** (DMSO-d_6, 299 K, 500 MHz) S13
HMBC spectrum of synthetic **1** (DMSO-d_6, 298 K, 500 MHz) S14

Ethyl 2-methyl-3-oxo-3-(phenylamino)propanoate

UV spectrum S15
IR spectrum S16
^{1}H NMR spectrum ($CDCl_3$, 500 MHz) S17
^{13}C NMR spectrum ($CDCl_3$, 125 MHz) S18

N-Phenyl-2-methylmalonamic acid

UV spectrum S19
IR spectrum S20
^{1}H NMR spectrum (DMSO-d_6, 500 MHz) S21
^{13}C NMR spectrum (DMSO-d_6, 125 MHz) S22

Synthetic procedure of 1

Compound **1** was synthesized according to the procedure (Scheme S1) established by Kawada and coworkers [1].

Diethyl methyl malonate (220 mg, 1.26 mmol, 1.0 equiv) was dissolved in THF (1.4 mL) and 0.25 M KOH (6.28 mL，157 mmol, 1.25 equiv) was added to the solution dropwise at 0 °C. After stirring at room temperature for 2 h, the reaction solution was acidified with 1 M HCl (pH 3.0) and extracted with EtOAc. The combined organic layers were dried over anhydrous $MgSO_4$, filtered, and evaporated to give 2-methylmalonic acid ethyl ester (186.2 mg, 1.10 mmol, 87%).

To a stirring solution of 2-methylmalonic acid ethyl ester (186.2 mg, 1.10 mmol, 1.0 equiv) in CH_2Cl_2 (2 mL) were added oxalyl chloride (200 µL, 1.68 mmol, 1.5 equiv) and a few drops of DMF, and the resulting mixture was kept stirred for 3 h at ambient temperature. After concentration, the generated ethyl 2-methylmalonyl chloride (166.1 mg, 1.0 mmol, 1.0 equiv) was dissolved in CH_2Cl_2 (1.5 mL) and condensed with aniline (66 µL, 1.0 mmol, 1.0 equiv) under the catalysis of Et_3N (150 µL) first at 0 °C, and then at room temperature over 6 h. The reaction was quenched with 0.1 M HCl (500 µL) and extracted with CH_2Cl_2 (500 µL). The CH_2Cl_2 layer was washed with saturated $NaHCO_3$ and NaCl solution, dried over anhydrous $MgSO_4$, filtered, and evaporated to give of ethyl 2-methyl-3-oxo-3-(phenylamino)propanoic acid (176.5 mg, 0.72 mmol, 72%).

Ethyl 2-methyl-3-oxo-3-(phenylamino)propanoic acid: yellow powder; UV (MeCN) λ_{max} 242 nm (ε 13900); IR (ATR) ν_{max} 3308, 3203, 2984, 2940, 2880, 1738, 1661, 1599, 1543, 1499, 1442, 1245, 1208, 1174, 1095, 1078, 933, 861, 753, 691 cm^{-1}; ^1H NMR (500 MHz, $CDCl_3$), δ 1.32 (3H, t, $J_{5,4}$=7.2 Hz), 1.56 (3H, d, $J_{6,2}$=7.3 Hz), 3.45 (1H, q, $J_{2,6}$=7.3 Hz), 4.26 (2H, q, $J_{4,5}$=7.2 Hz), 7.13 (1H, t, $J_{4',3'\ (4',3'')}$=7.4 Hz), 7.34 (2H, dt, $J_{3',4'\ (3'',4')}$=7.4 Hz, $J_{3',2'\ (3'',2'')}$=7.7 Hz), 7.55 (2H, d, $J_{2',3'\ (2'',3'')}$=7.7 Hz), 8.63 (1H, br); ^{13}C NMR (125 MHz, $CDCl_3$), δ 14.1 (C-5), 15.5 (C-6), 47.5 (C-2), 61.9 (C-4), 120.0 (C-2', 2''), 124.5 (C-4'), 129.0 (C-3', 3''), 137.6 (C-1'), 166.9 (C-1), 173.0 (C-3).

To a stirring solution of ethyl 2-methyl-3-oxo-3-(phenylamino)propanoic acid in a sealed tube was first added a mixture of 0.5M KOH/THF (1:1, 9.6 mL) at 0 °C and the mixture was stirred at room temperature for 1 h. Then, the mixture was acidified with 1 M HCl (pH 4.0) and extracted with EtOAc, dried over anhydrous $MgSO_4$, filtered, and evaporated to give a white powder of *N*-phenyl-2-methylmalonamic acid (96.5 mg, 0.45 mmol, 63%).

N-phenyl-2-methylmalonamic acid: UV (MeCN) λ_{max} 242 nm (ε 13300); IR (ATR) ν_{max} 3332, 3145, 2990, 2945, 2887, 1744, 1627, 1595, 1550, 1499, 1445, 1284, 1215, 1170, 1098, 1079, 932, 868, 751, 692 cm^{-1}; ^1H NMR (500 MHz, DMSO-d_6), δ 1.27 (3H, d, $J_{4,2}$=7.1 Hz), 3.50 (1H, q, $J_{2,4}$=7.1 Hz), 7.04 (1H, t, $J_{4',3'\ (4',3'')}$=7.4 Hz), 7.30 (2H, dt, $J_{3',4'\ (3'',4')}$=7.4 Hz, $J_{3',2'\ (3'',2'')}$=7.5 Hz), 7.60 (2H, d, $J_{2',3'\ (2'',3'')}$=7.5 Hz), 10.1 (1H, br); ^{13}C NMR (125MHz, DMSO-d_6), δ 14.3 (C-4), 47.4 (C-2), 120.0 (C-2', 2''), 123.8 (C-4'), 129.2 (C-3', 3''), 139.5 (C-1'), 169.0 (C-1), 172.4 (C-3).

N-Phenyl-2-methylmalonamic acid (63 mg, 0.32 mmol, 1.0 equiv) and Eaton's reagent (7.7 wt % phosphorus pentoxide in methanesulfonic acid, 400 μL, 0.1 equiv) were heated at 85 °C in a sealed tube with stirring for 2.5 h. After cooling to room temperature, the reaction mixture was neutralized with $NaHCO_3$ powder, filtered, and dried in vacuo. The residue was dissolved in 16% MeCN/0.1% HCOOH for one night and subjected to preparative HPLC with isocratic elution with the same solvent mixture to give **1** (10.3 mg, 0.06 mmol, 19%) at t_R 31 min.

Synthetic 4-hydroxy-3-methyl-2(1*H*)-quinolone (**1**): UV (MeOH) λ_{max} nm (ε): 312 (2200), 280 (1800), 223 (10200); IR ν_{max} (ATR) cm^{-1} 3364, 2821, 2734, 1580, 1494, 1381, 1349, 1268, 750, 687, 670, 664; HR-ESITOFMS *m/z* 198.0525 $[M+Na]^+$ (calcd for $C_{10}H_9NNaO_2$, 198.0526); ^1H and ^{13}C NMR data in DMSO-d_6 are essentially the same to natural **1**.

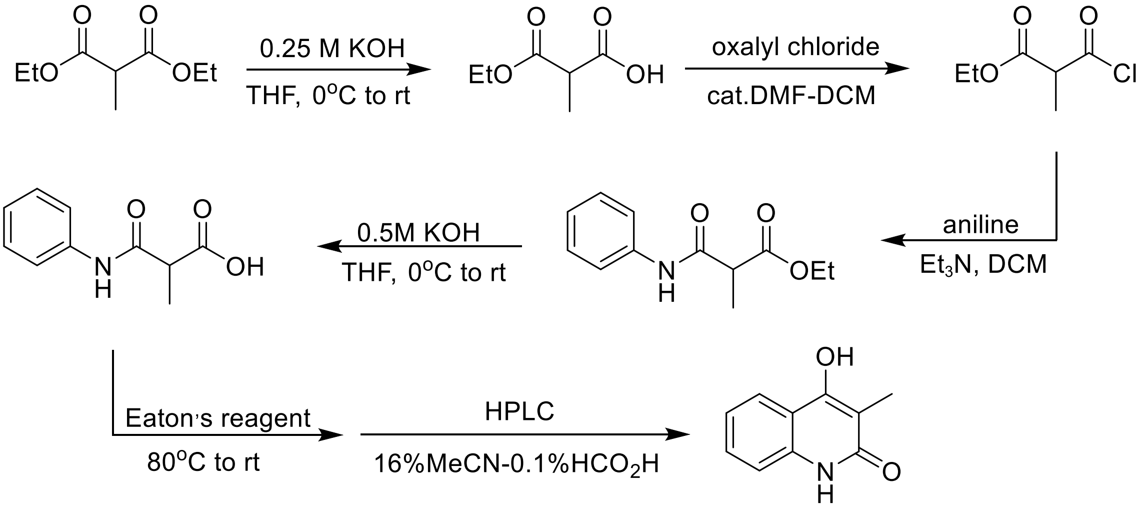

1

Scheme S1: Total synthesis of **1**.

References

1. Kawada, M.; Inoue, H.; Ohba, S.; Hatano, K.; Abe, H.; Hayashi, C.; Watanabe, T.; Igarashi, M. PCT Int. Appl. WO 2014/132902 A1.

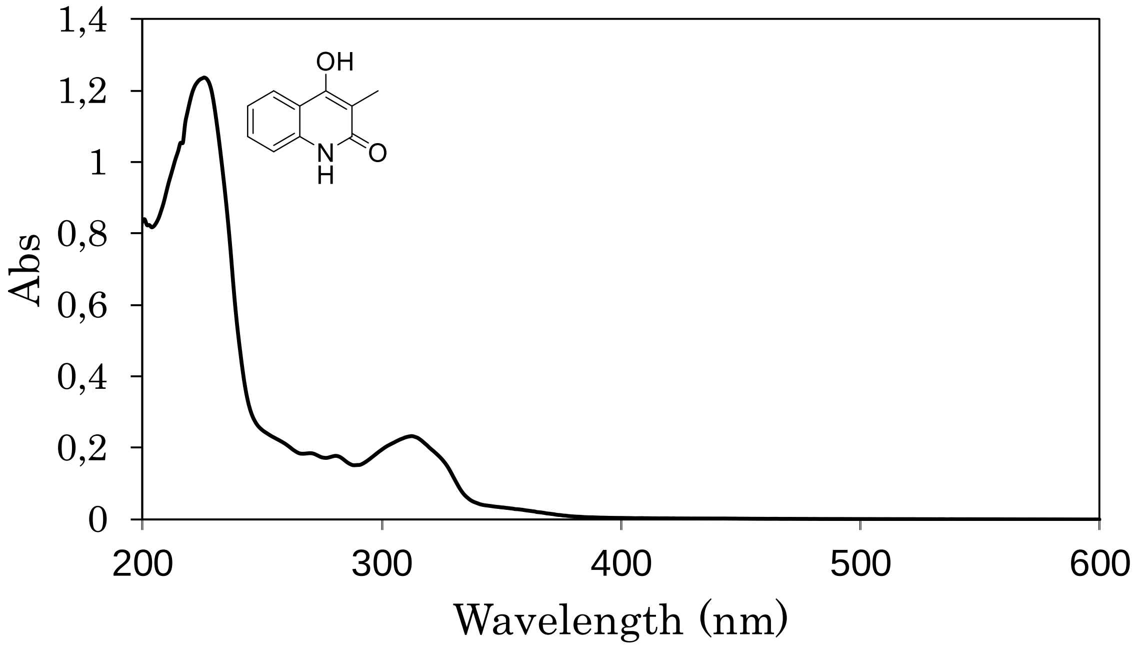

UV spectrum of natural **1**

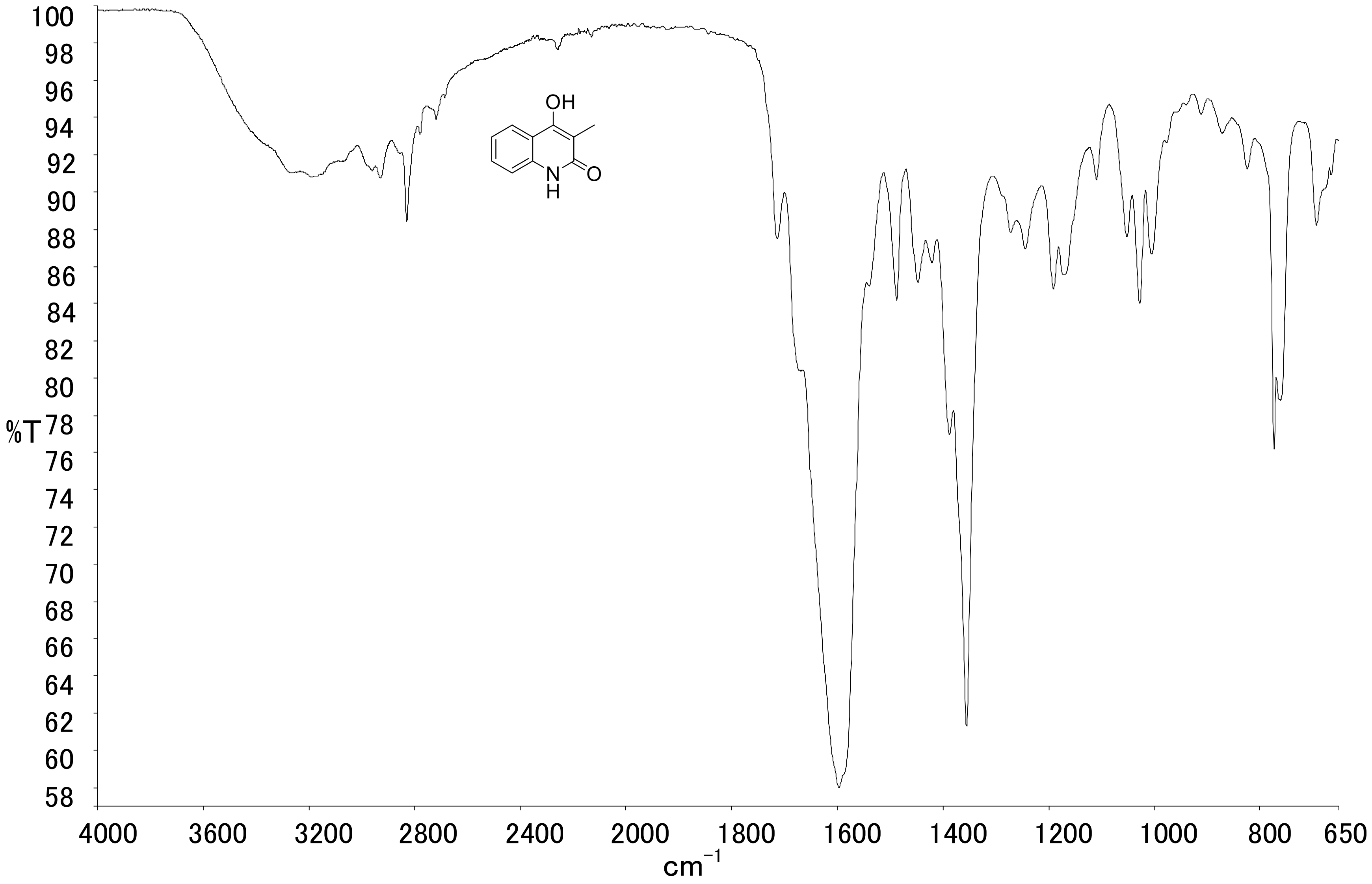

IR spectrum of natural **1**

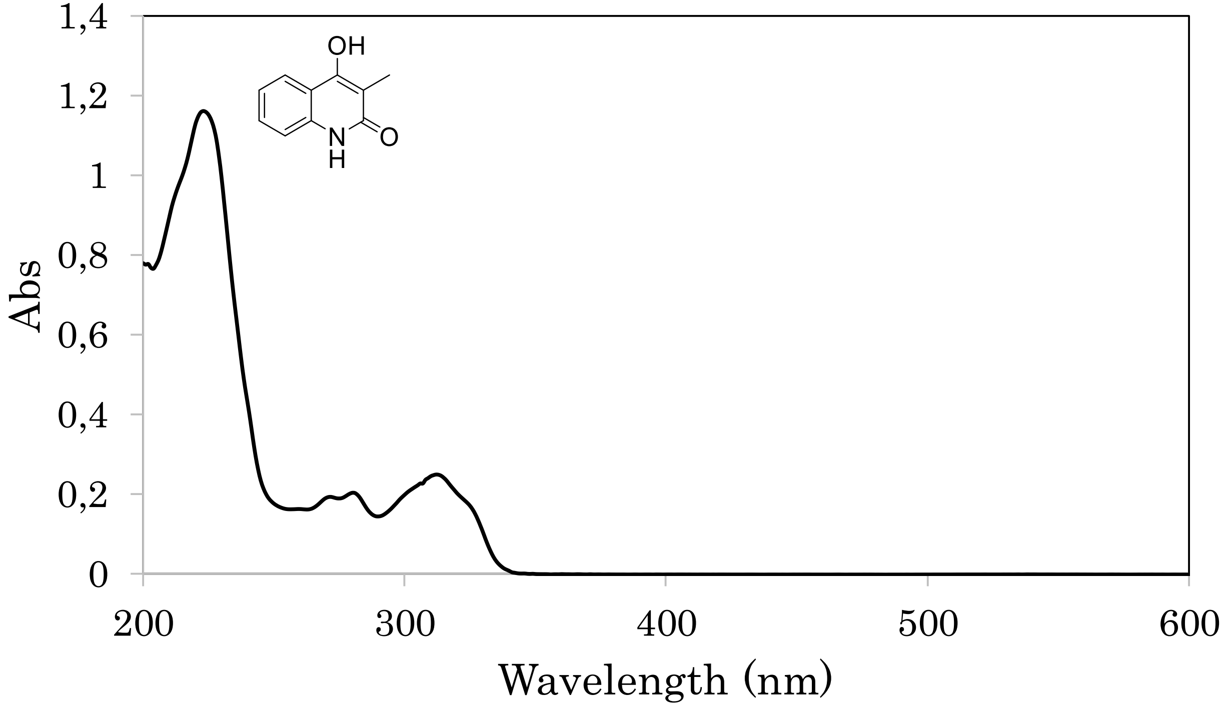

UV spectrum of synthetic **1**

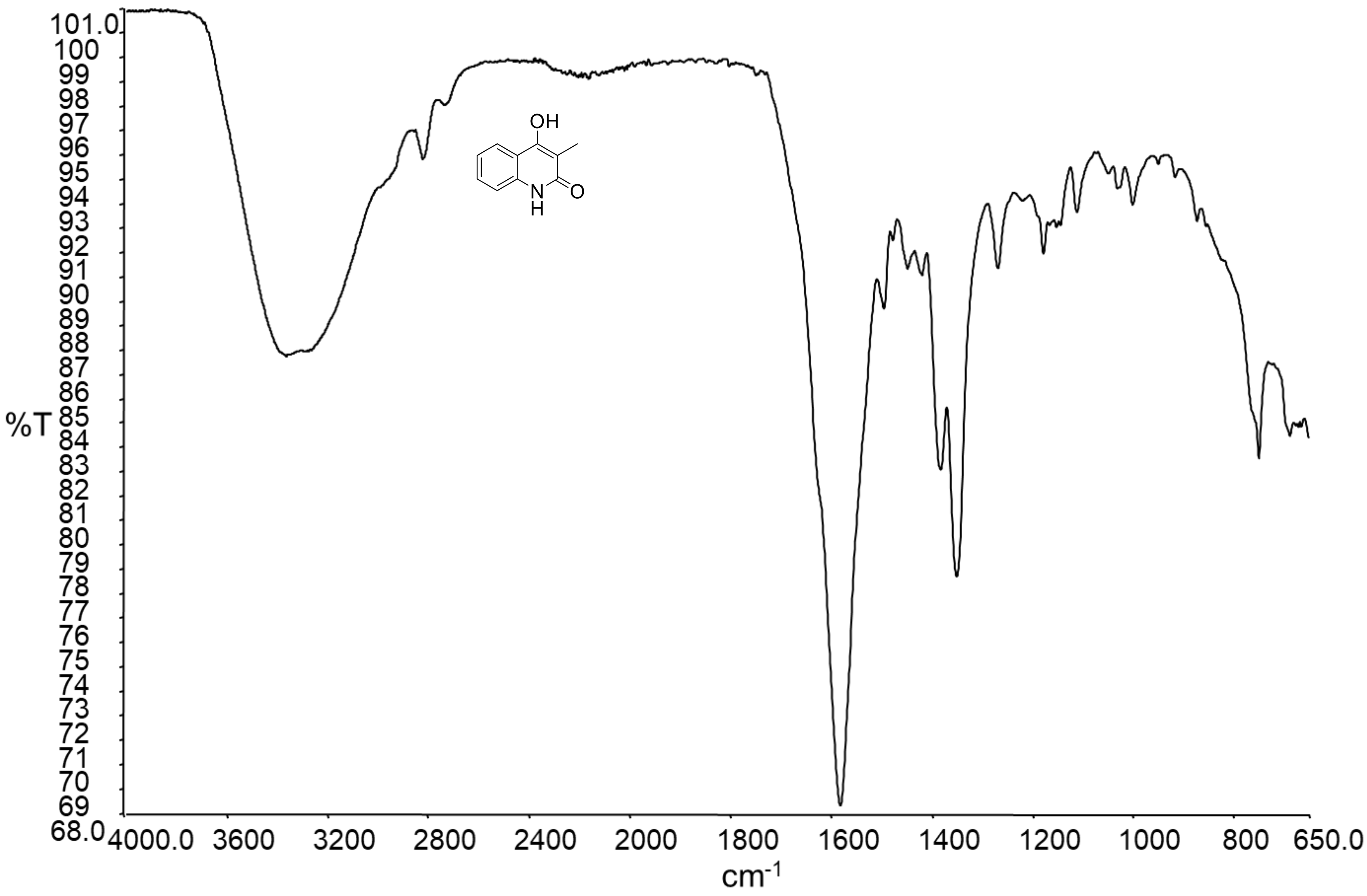

IR spectrum of synthetic **1**

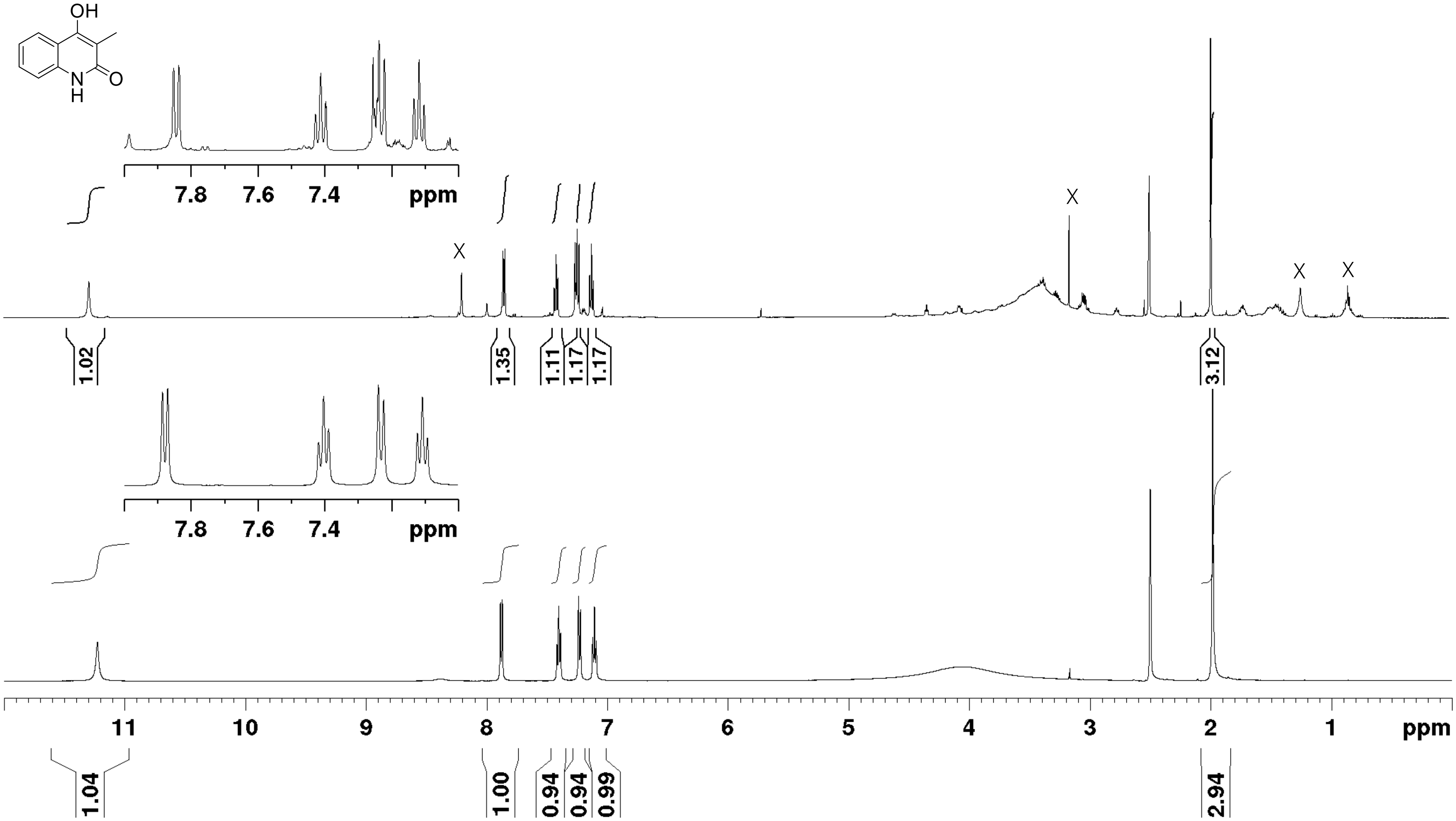

^{1}H NMR spectra of natural (upper) and synthetic (lower) **1** (500 MHz, 297 K, DMSO-d_6)

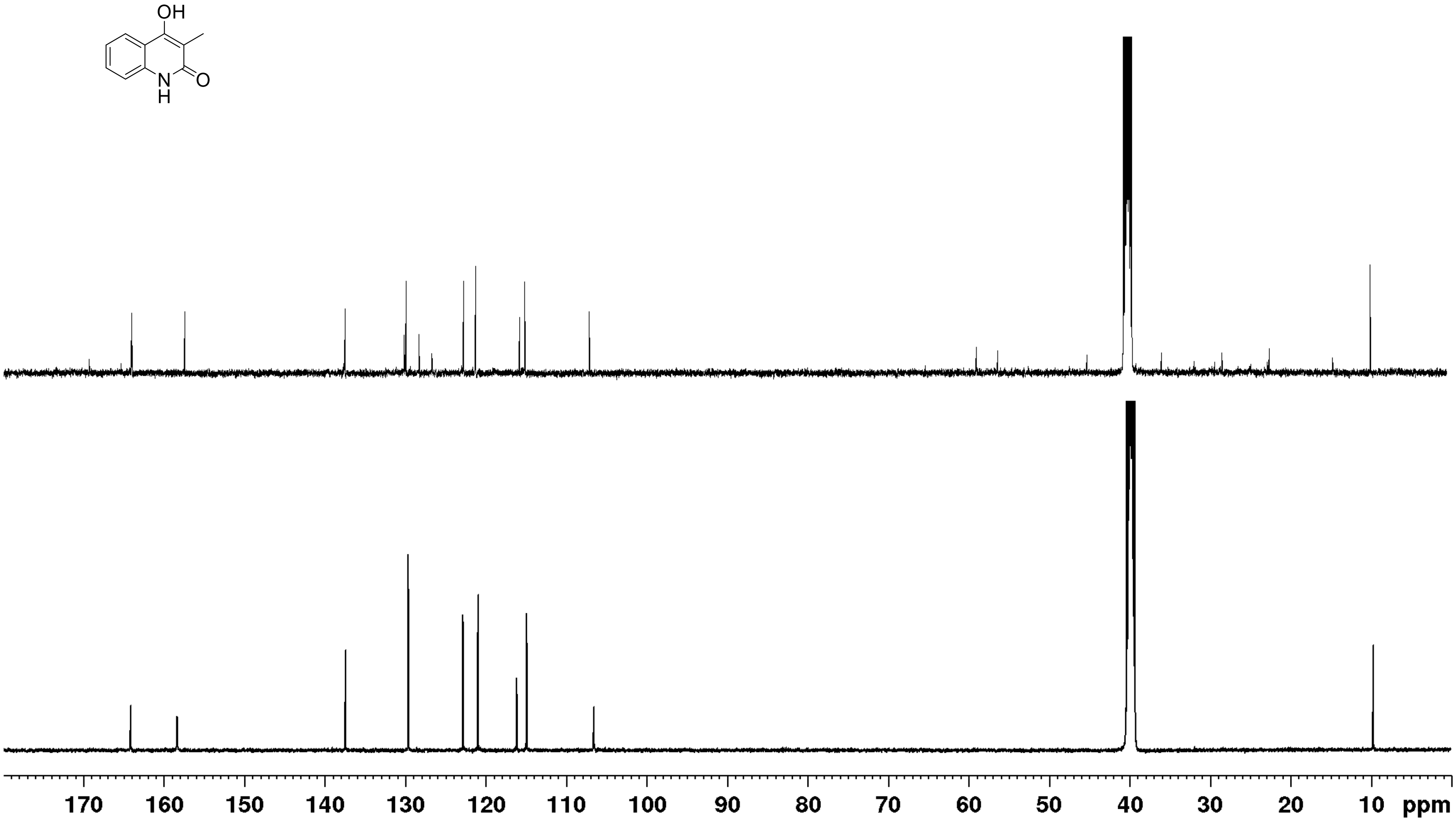

^{13}C NMR spectra of natural (upper) and synthetic (lower) **1** (125 MHz, 297 K, DMSO-d_6)

.

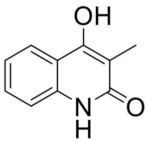

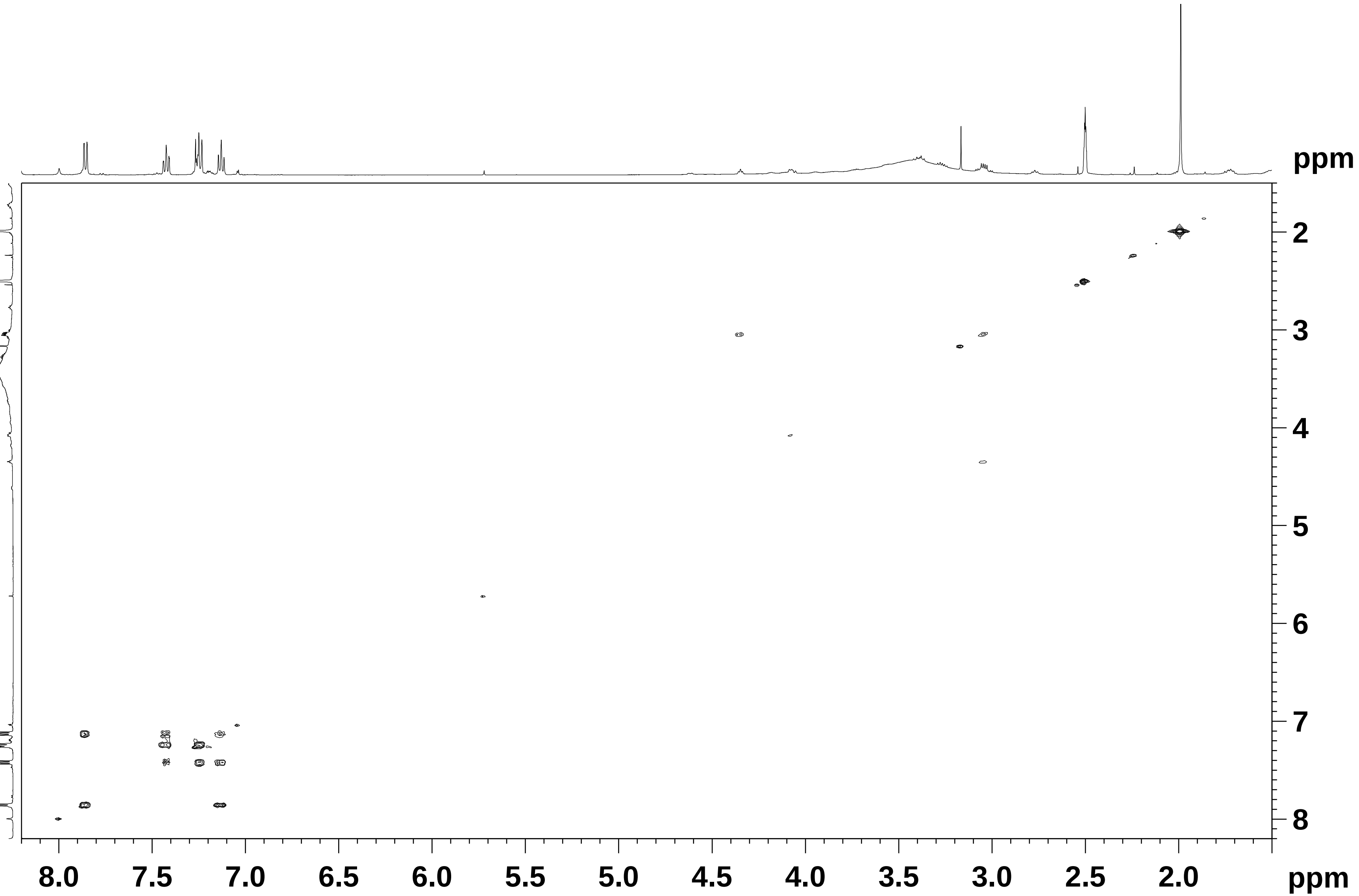

COSY spectrum of natural **1** (500 MHz, 297 K, DMSO-d_6)

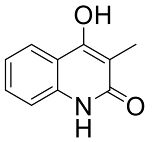

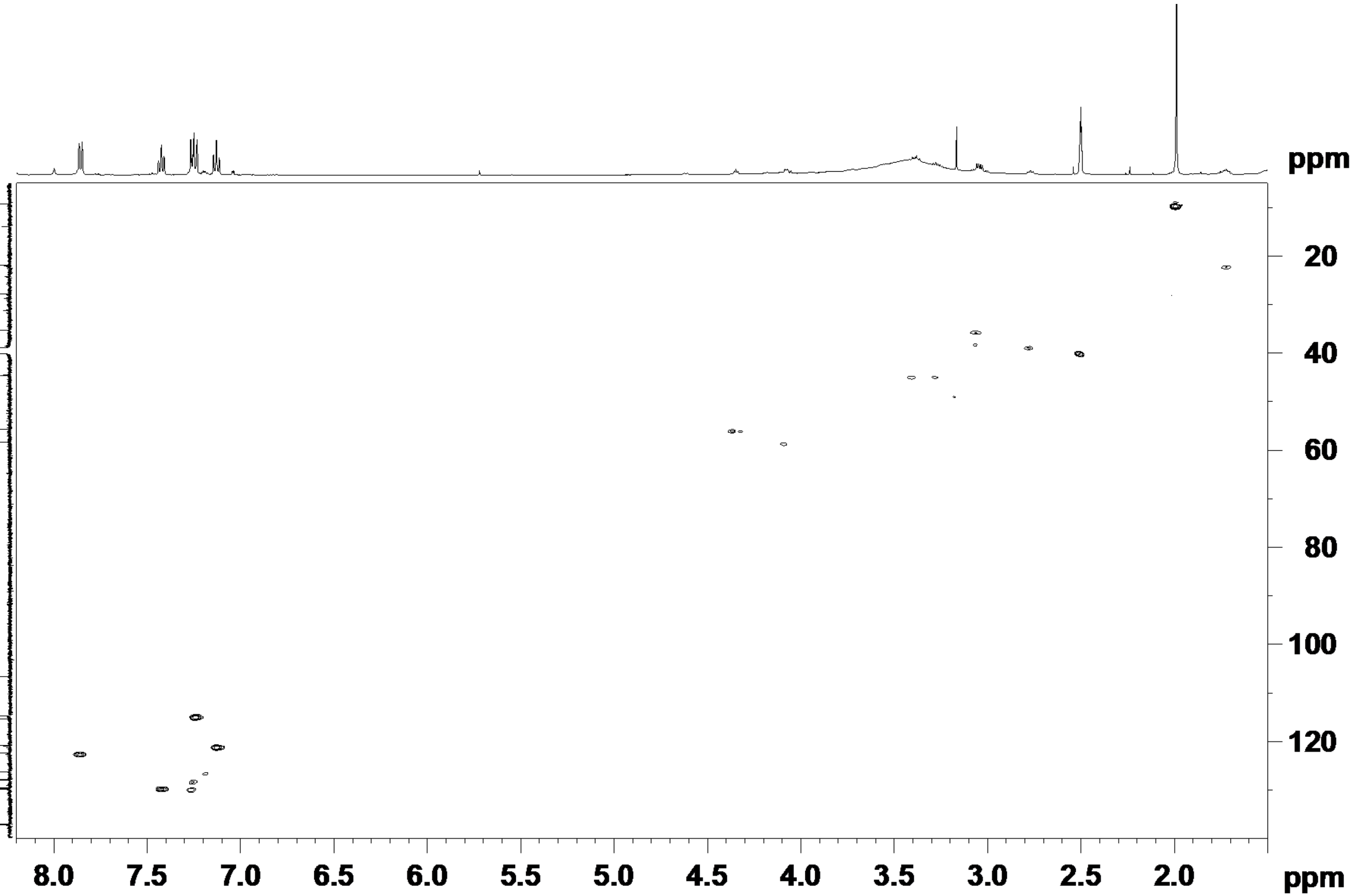

HSQC spectrum of natural **1** (500 MHz, 297 K, DMSO-*d*6)

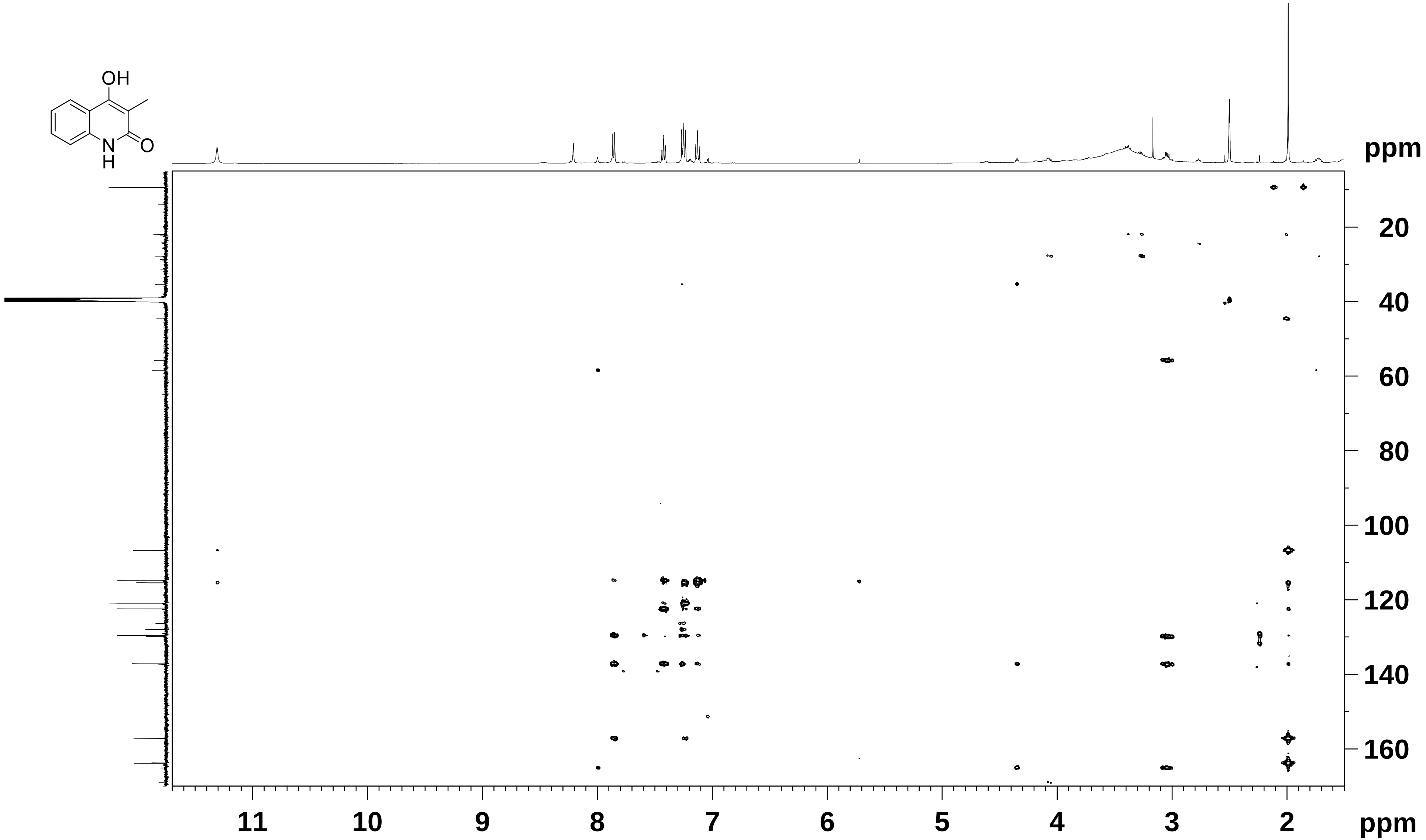

HMBC spectrum of natural **1** (500 MHz, 298 K, DMSO-d_6)

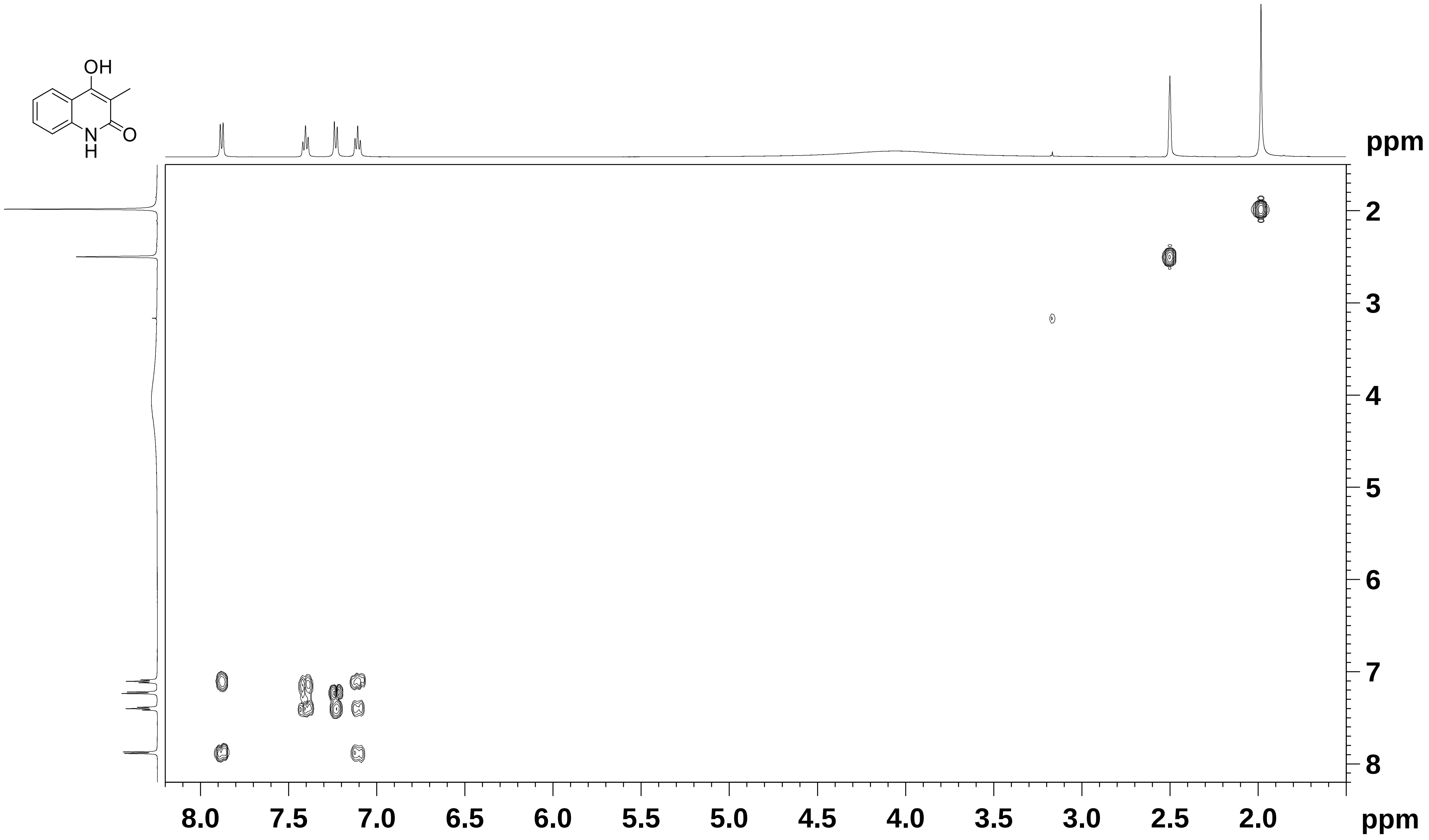

COSY spectrum of synthetic **1** (500 MHz, 298 K, DMSO-d_6).

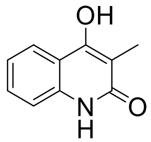

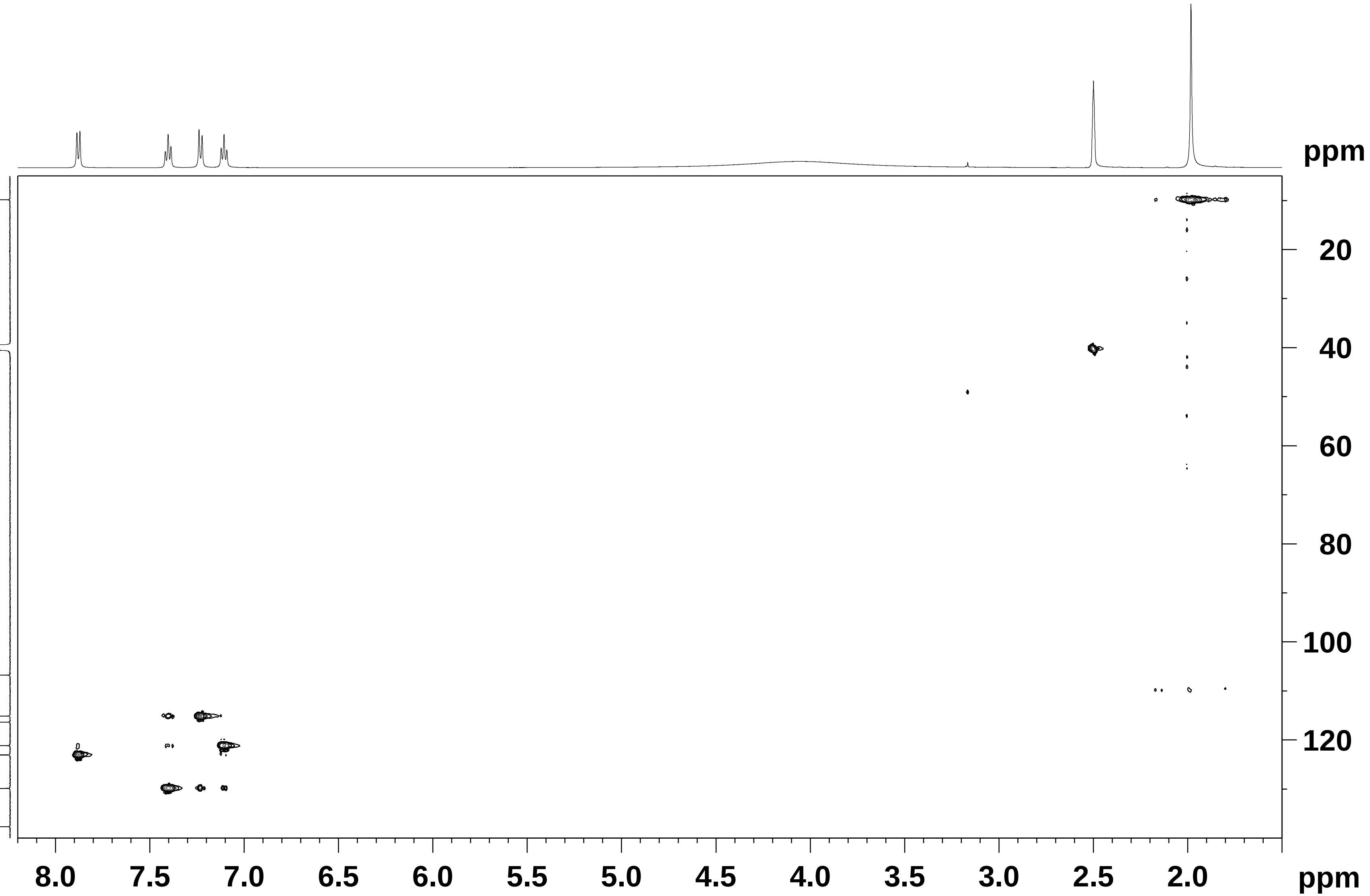

HSQC spectrum of synthetic **1** (500 MHz,299 K, DMSO-*d*6).

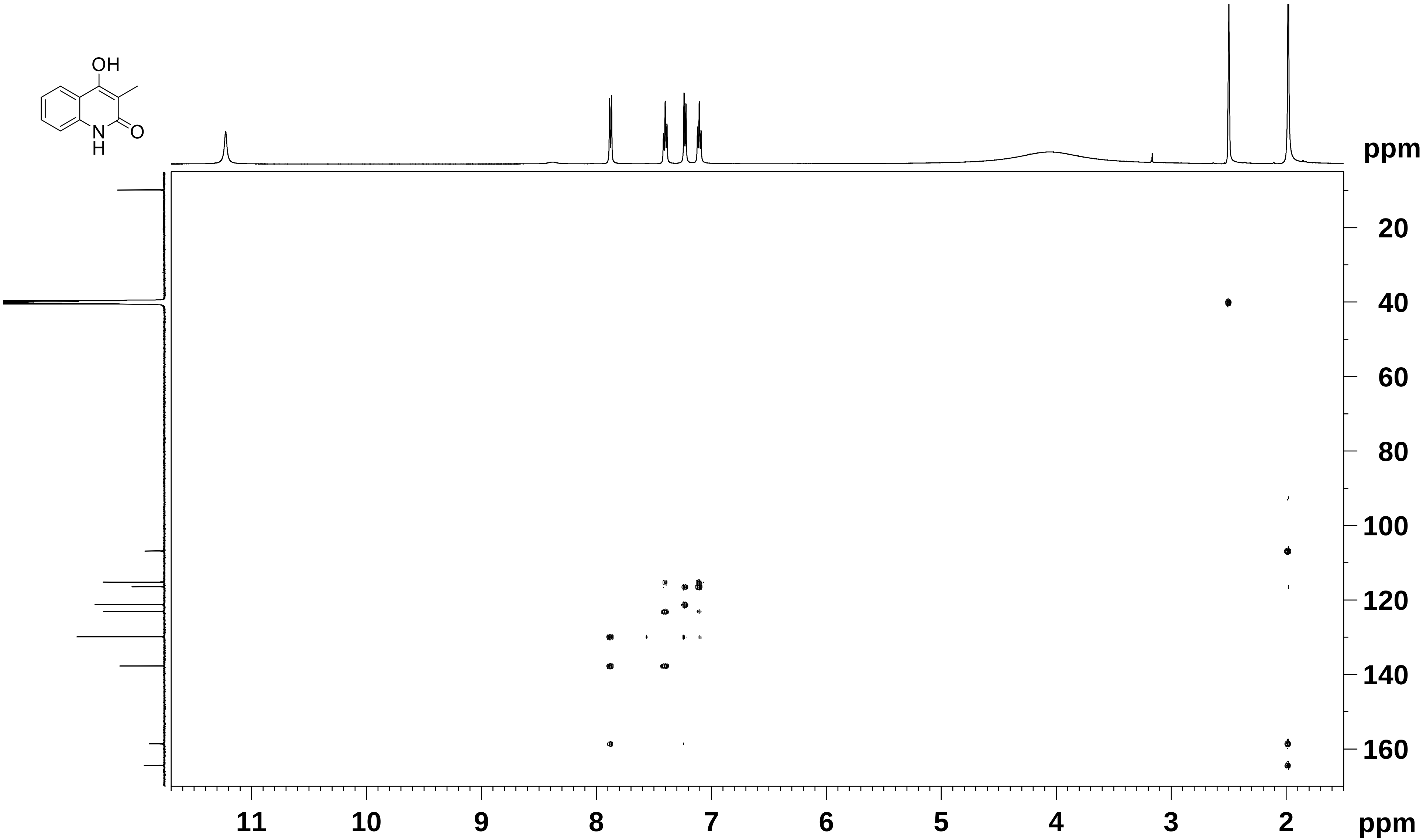

HMBC spectrum of synthetic **1** (500 MHz, 298 K, DMSO-d_6).

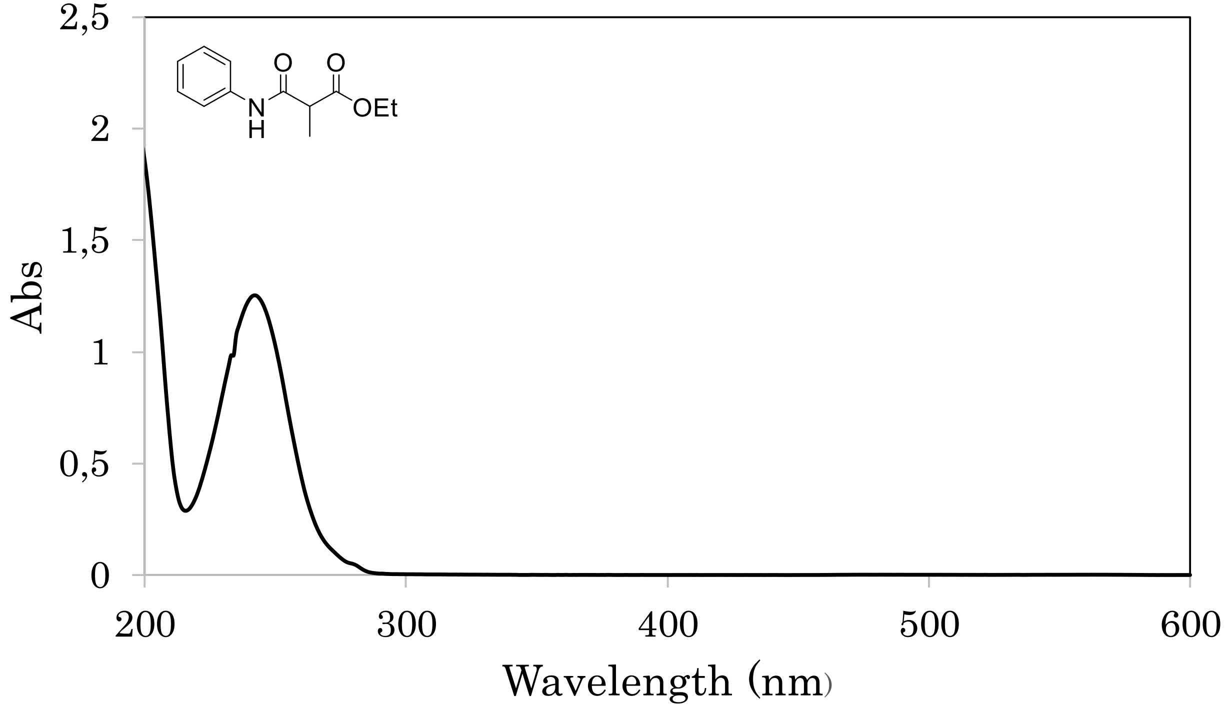

UV spectrum of ethyl 2-methyl-3-oxo-3-(phenylamino)propanoic acid

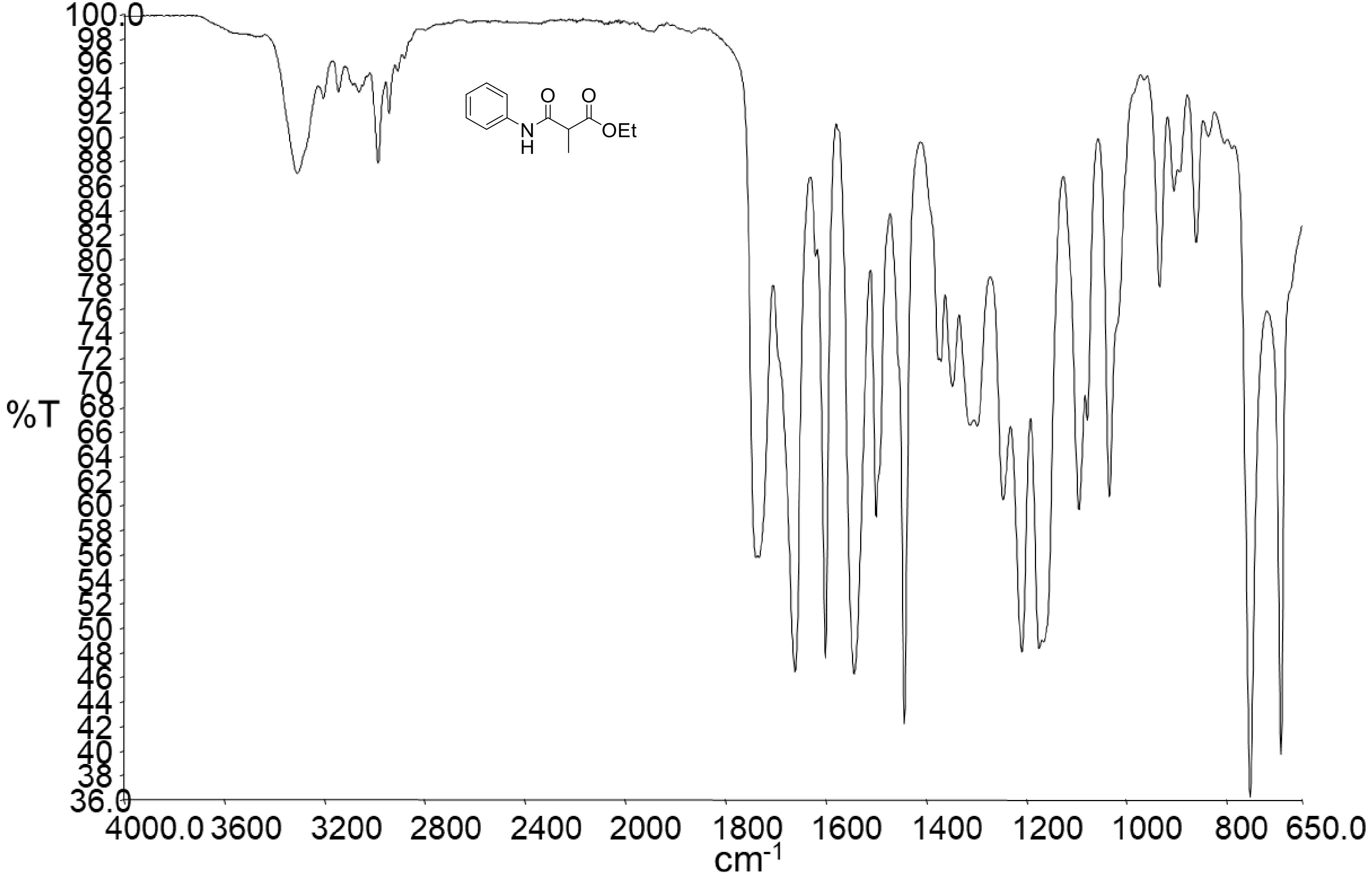

IR spectrum of ethyl 2-methyl-3-oxo-3-(phenylamino)propanoic acid

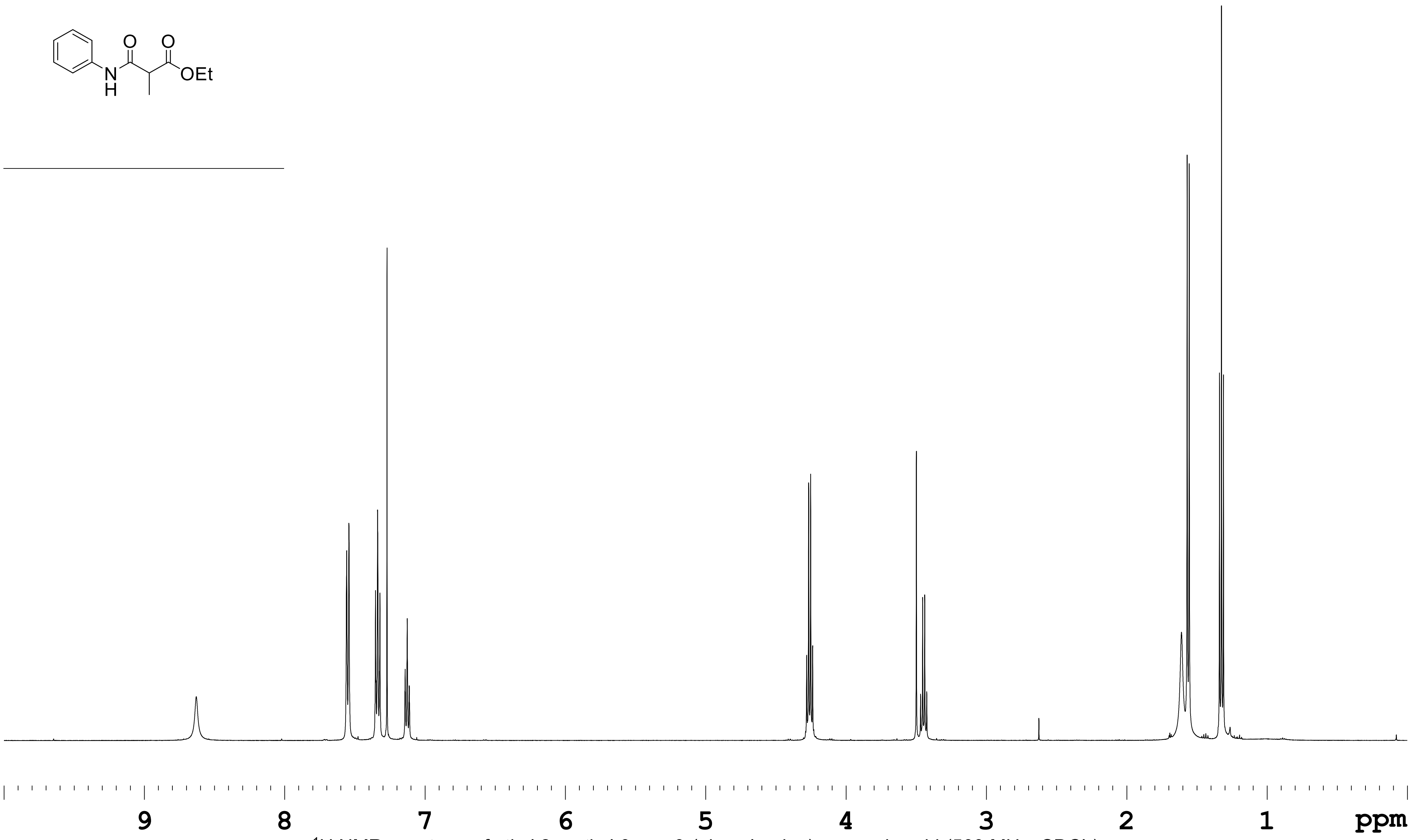

^{1}H NMR spectrum of ethyl 2-methyl-3-oxo-3-(phenylamino)propanoic acid (500 MHz, $CDCl_3$)

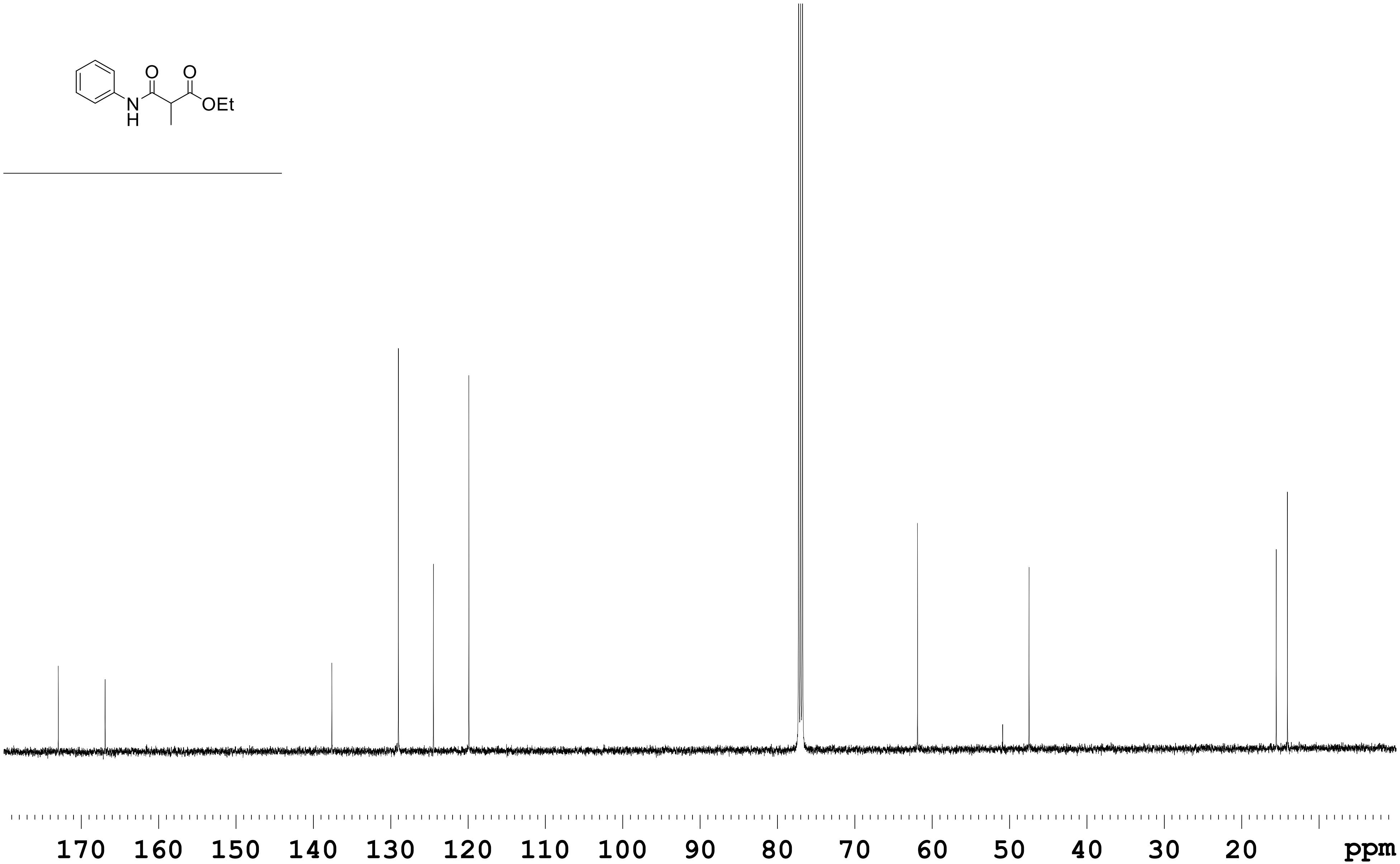

^{13}C NMR spectrum of ethyl 2-methyl-3-oxo-3-(phenylamino)propanoic acid (125 MHz, $CDCl_3$)

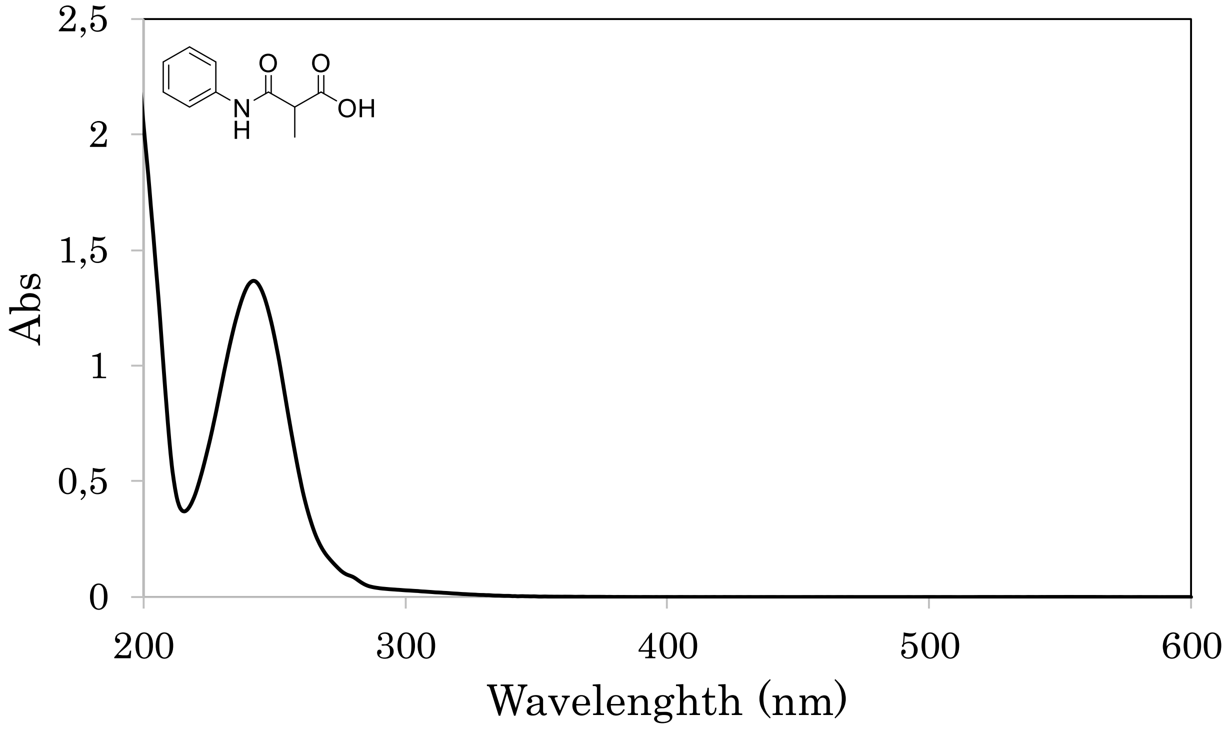

UV spectrum of *N*-phenyl-2-methylmalonamic acid

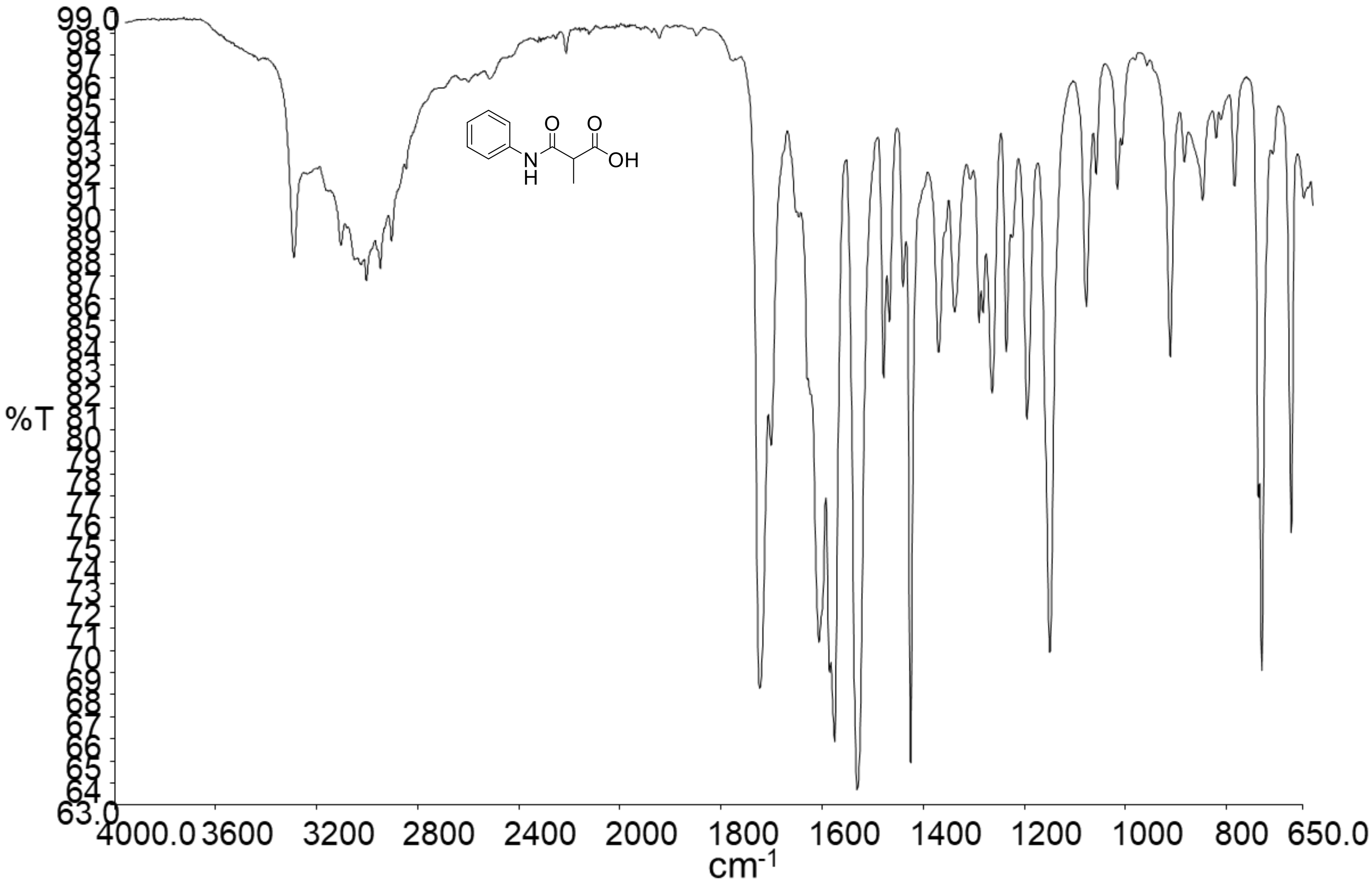

IR spectrum of *N*-phenyl-2-methylmalonamic acid

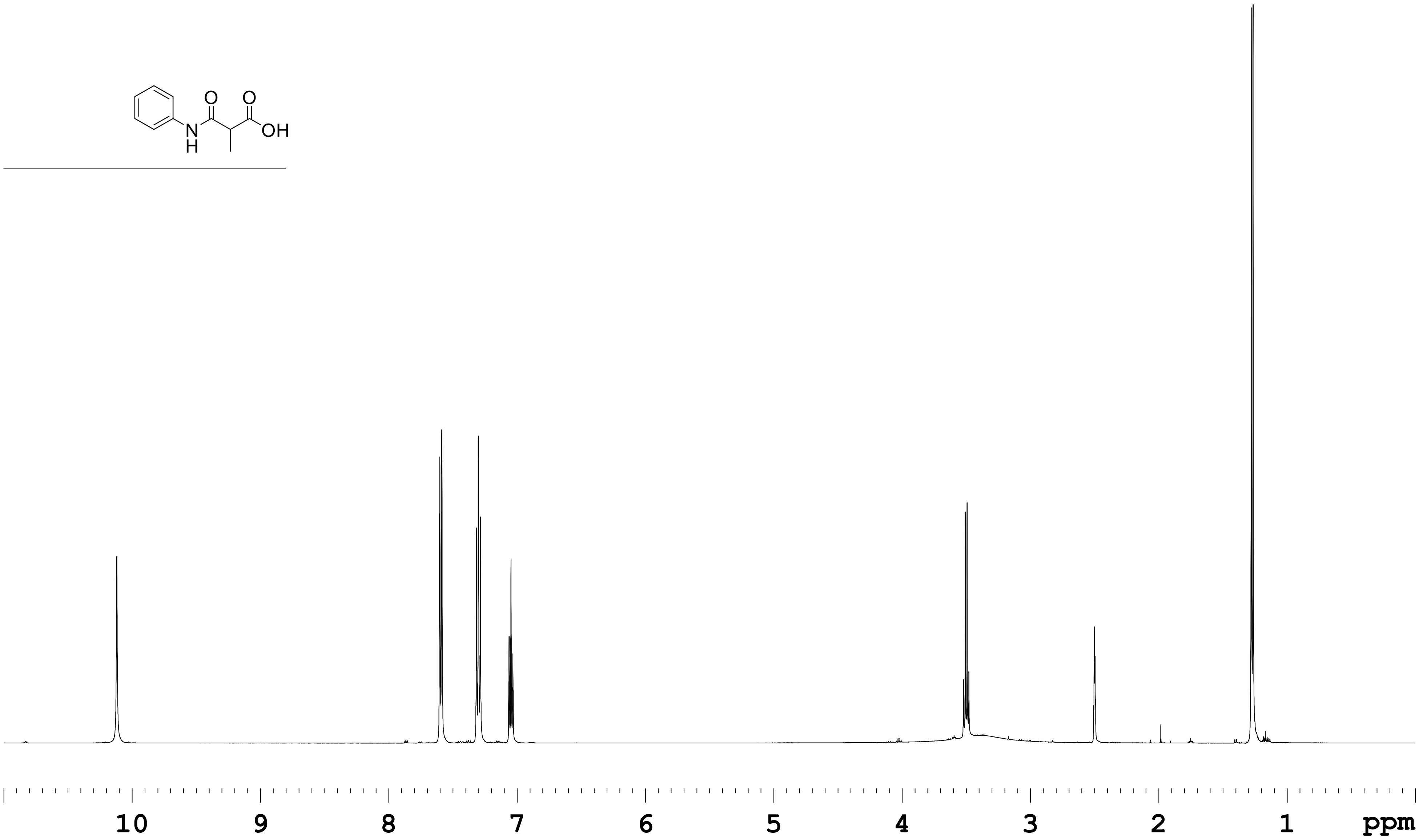

^{1}H NMR spectrum of *N*-phenyl-2-methylmalonamic acid (500 MHz, DMSO-d_6)

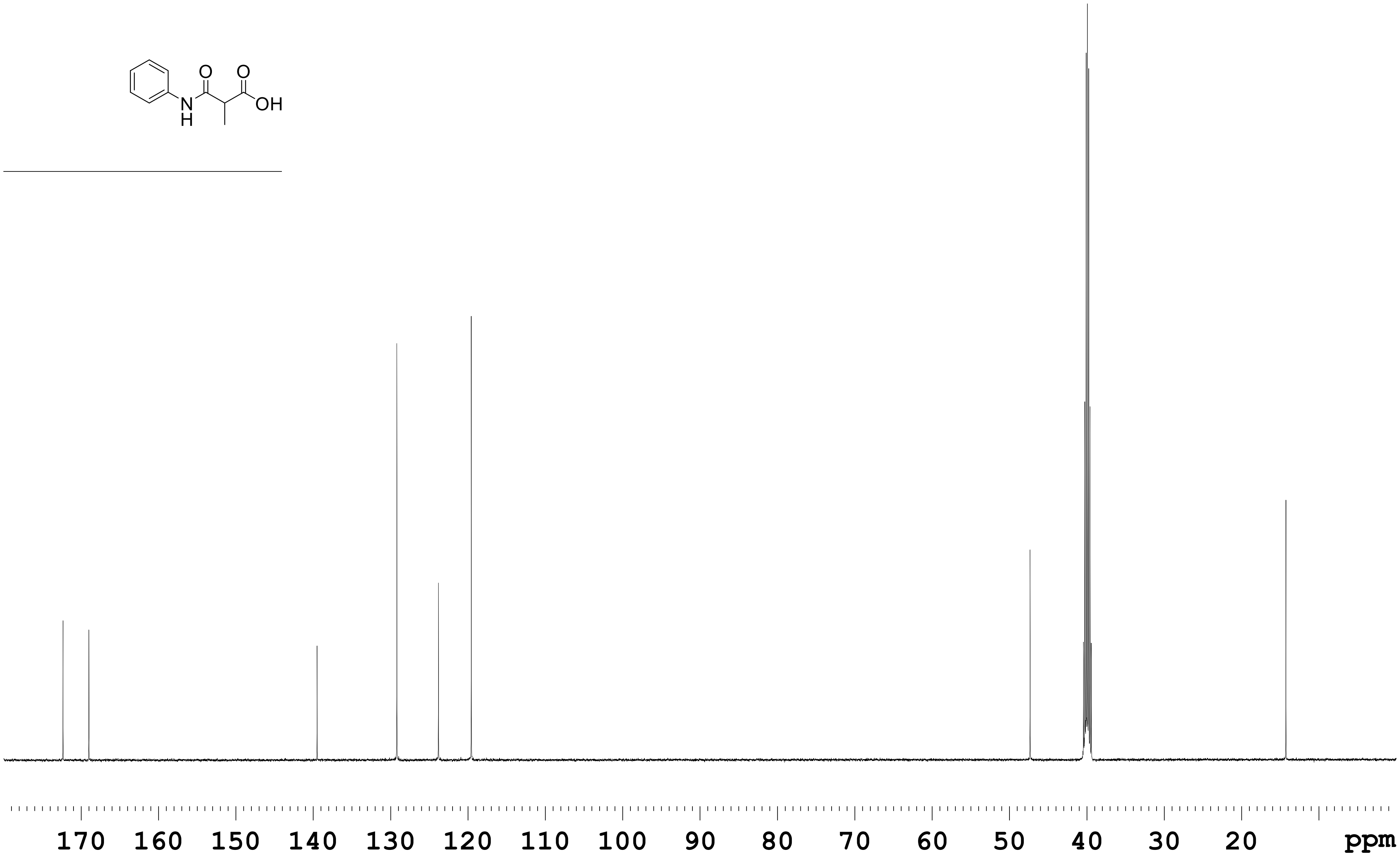

^{13}C NMR spectrum of *N*-phenyl-2-methylmalonamic acid (125 MHz, DMSO-d_6)
